# Evolution of RGF/GLV/CLEL Peptide Hormones and Their Roles in Land Plant Growth and Regulation

**DOI:** 10.3390/ijms222413372

**Published:** 2021-12-13

**Authors:** Yitian Fang, Jinke Chang, Tao Shi, Wenchun Luo, Yang Ou, Dongshi Wan, Jia Li

**Affiliations:** 1 State Key Laboratory of Microbial Metabolism, School of Life Sciences and Biotechnology, Shanghai Jiao Tong University, Shanghai 200240, China; fangyt@sjtu.edu.cn; 2 Ministry of Education Key Laboratory of Cell Activities and Stress Adaptations, School of Life Sciences, Lanzhou University, Lanzhou 730000, China; kingco_chang@163.com (J.C.); luowch12@lzu.edu.cn (W.L.); ouy@lzu.edu.cn (Y.O.); 3 CAS Key Laboratory of Aquatic Botany and Watershed Ecology, Wuhan Botanical Garden, Chinese Academy of Sciences, Wuhan 430074, China; shitao323@wbgcas.cn; 4 School of Life Sciences, Guangzhou University, Guangzhou 510006, China

**Keywords:** RGF, GLV, CLEL, peptide hormone, plant terrestrialization, root waving

## Abstract

Rooting is a key innovation during plant terrestrialization. RGFs/GLVs/CLELs are a family of secreted peptides, playing key roles in root stem cell niche maintenance and pattern formation. The origin of this peptide family is not well characterized. *RGFs* and their receptor genes, *RGIs*, were investigated comprehensively using phylogenetic and genetic analyses. We identified 203 *RGF* genes from 24 plant species, representing a variety of land plant lineages. We found that the *RGF* genes originate from land plants and expand via multiple duplication events. The lineage-specific *RGF* duplicates are retained due to their regulatory divergence, while a majority of *RGFs* experienced strong purifying selection in most land plants. Functional analysis indicated that *RGFs* and their receptor genes, *RGIs*, isolated from liverwort, tomato, and maize possess similar biological functions with their counterparts from Arabidopsis in root development. *RGFs* and *RGIs* are likely coevolved in land plants. Our studies shed light on the origin and functional conservation of this important peptide family in plant root development.

## 1. Introduction

The development of a robust root system is one of the essential innovations during plant terrestrialization. The terrestrial environment differs from the aquatic environment because water and soluble minerals are disproportionately distributed in the soil due to gravity and evaporation [1]. Therefore, during the early colonization of land, the development of a root system is a giant leap, enabling plants to anchor and uptake water and nutrients in the soil [2,3]. Root initiation and architecture optimization follow with the whole process of land plant radiation [4]. Generally, based on whether they possess a root system, plants can be divided into two types. The first type is named vascular plants, which contain recognizable roots with an apical meristem and a root cap to protect the apex of the growing root [5]. Another type is non-vascular plants, such as mosses and liverworts, which contain rhizoids without root caps. Studies showed that the roots in plants were evolved in a step-wise fashion, from rhizoids to roots with meristem, and to roots with root cap or root hair, as observed in vascular land plants [5]. However, the evolution of crucial genetic mechanisms underpinning the root formation and gravitropic response of land plants, particularly mosses and liverworts, needs to be investigated.

Plant peptide hormones play key roles in various processes during plant growth, development, and stress adaptations, such as cell proliferation and differentiation, stomatal development, self-incompatibility, and defense responses [6,7]. Root meristem growth factor/GOLVEN/CLE-like (RGF/GLV/CLEL) peptides are a group of post-translationally modified and secreted signal molecules. They were identified in Arabidopsis by three independent research groups with different strategies [8,9,10]. Chemically synthesized mature RGF peptides restored the meristematic activity of a tyrosylprotein sulfotransferase mutant, *tpst-1*, in the presence of additional two sulfated peptides, PSK and PSY1. They were initially named RGFs. Because a motif in the C-terminus of CLE18 is similar to RGF peptides, a different research group defined this family as a CLE-like family [9]. They were also named GOLVENs because they can cause irregular root waving when overexpressed, possibly due to the reduction in gravitropism [10].

Up to now, a total of 11 RGFs have been identified in Arabidopsis that are involved in plant development, such as root meristem maintenance, root hair development, lateral root development, and root and hypocotyl gravitropism [8,9,10]. Similar to other secreted signal peptides, mature RGF polypeptides are processed by proteolytic cleavages from their preproproteins. A precursor protein usually contains approximately 79–182 amino acids with an N-terminal signal peptide that directs it into the secretory pathway and a C-terminal conserved RGF domain [8,9,10]. The mature peptides contain 13–16 amino acid residues, which usually require post translational modifications, including tyrosine sulfation and proline hydroxylation.

Previous studies showed that auxin can up-regulate the expression of *TPST* [11]. AtRGF1 can be sulfated by TPST. Correctly modified RGFs can be perceived by RGIs/RGFRs [12,13,14], a group of leucine-rich repeat receptor-like kinases, up-regulating the two downstream transcription factor genes *PLATHOLA1* (*PLT1*) and *PLT2*, thereby mediating the development of apical meristem. RGIs/RGFRs were identified by three independent research groups using different approaches [12,13,14]. Studies showed that *rgi1*,*2*,*3*,*4*,*5* quintuple mutant exhibits a severe short root phenotype with reduced meristem size and a complete insensitivity to exogenously applied AtRGF1. Dot-blotting and protein interaction analysis indicated that the extracellular domain of AtRGI1 is able to bind AtRGF1 directly. AtRGF1 can induce autophosphorylation and ubiquitination of its receptors, such as AtRGI1 and AtRGI2 [12].

Many *RGF* genes are expressed in roots, but some members are also expressed in shoots, reproductive organs, and even in specific cells or cell types [15,16], suggesting that functions of RGFs are diversified during plant evolution. There are indeed comprehensive functional studies of RGF peptides in the eudicot model plant—Arabidopsis. Nevertheless, the evolution and functions of RGF peptides in other major clades, particularly early diverging land plants, are not characterized. To better understand how evolution of *RGF* gene family contributes to the root development that helps plant to conquest the landing, we first comprehensively analyzed the *RGFs* for their key molecular features such as signal peptides, chemical properties, gene structures, and conserved motifs, etc. We then performed evolutionary analyses including phylogeny, sequence clustering, selection pressure, and tissue expression profiling. It is noteworthy that we also studied the molecular genetic mechanisms and evolution of RGFs and their receptors, RGIs, in representative plant species, which could trace their common ancestor of more than 300 million years ago. Our findings establish that the members of RGF/RGI ligand–receptor system have been conserved since the emergence of liverworts, providing a framework for further evolutionary and functional study of the signaling pathways triggered by RGF peptides in land plants.

## 2. Results

### 2.1. Emergence and Evolution of RGFs during Plant Terrestrialization

To explore the origin and evolution of RGF family members, we carefully selected 24 representative species, including green algae and land plants (Table S1). RGF members in these species were identified by a comprehensive homology search using BLAST and HMMER software, followed by removing the redundant genes and genes without an RGF domain. To test whether this peptide family is originated from horizontal gene transfer, we also searched the genomic sequences of a number of symbiotic bacteria (*Bradyrhizobium japonicum*, *Sinorhizobium meliloti*, *Mesorhizobium loti*), pathogenic bacteria (*Agrobacterium tumefaciens*, *Agrobacterium rhizogenes*), symbiotic fungi (*Laccaria bicolor*, *Glomus interadices*), and pathogenic fungi (*Botrytis cinerea*, *Ustilago maydis*, *Phytophthora sojae*). No *RGF*-like genes were found, suggesting that the RGF peptide family is indeed plant-specific.

**Figure 1:**
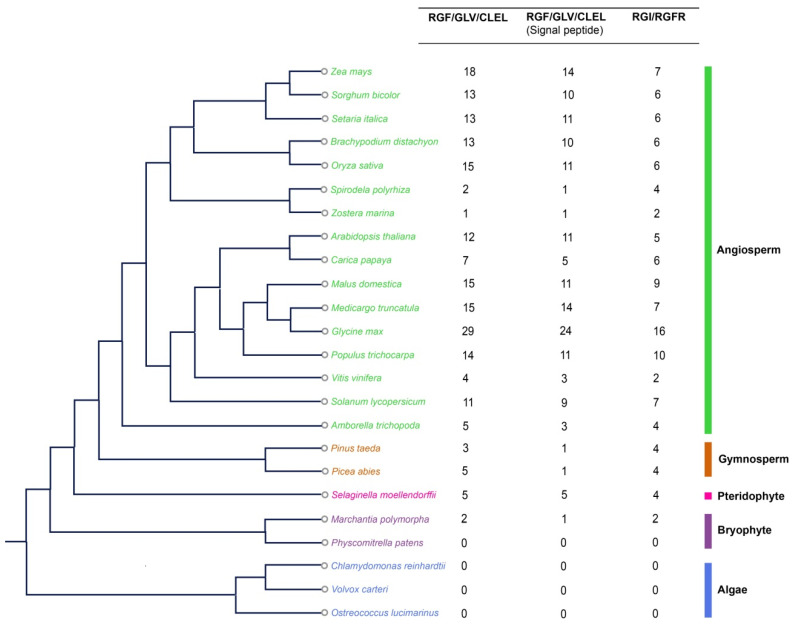
A summary of canonical RGF and RGI proteins identified in this study. The total number of RGF and RGI proteins identified in each plant genome is indicated in right. A total of 203 RGF candidate proteins from selected 24 species have been identified, and 158 of the 203 candidate proteins contain a typical signal peptide. AtCLE18, which contains both CLE and RGF domains, is not shown in this figure. The phylogenetic tree is modified from Phytozome (http://www.phytozome.net/).

A total of 203 RGF candidate proteins from these 24 species have been identified. We failed to identify any RGF members in algae but identified 2 RGFs and 2 RGIs in liverwort *Marchantia polymorpha* (Figure 1). Searching for the possible signal peptides in all identified RGFs using SignalP, we found that 158 of the 203 candidate proteins contain typical signal peptides. Only MpRGF1, but not MpRGF2, from *Marchantia polymorpha* contain a typical signal peptide. In *Arabidopsis thaliana*, 13 members have the RGF domain including AT3G60650 and AtCLE18 (Figure 1), in which AtCLE18 contains both CLE and RGF domains [9]. We compared the number of *RGF* genes to the size of their corresponding genome (Figure S1A) or to the total number of genes in the genome (Figure S1B). No apparent correlation was found between the number of identified *RGFs* and the size of their corresponding genome. For example, the maize genome is about 20 times larger than that of Arabidopsis, but it contains only a few more *RGF* genes than that of Arabidopsis. However, the number of *RGFs* is significantly correlated with the total number of genes in the genome, suggesting that RGF copy number and the total gene number evolve simultaneously. Intriguingly, we found a strong positive correlation between the number of *RGFs* and their receptors, *RGIs* (Figure S2), suggesting a coevolution of gene copy numbers between ligands and receptors. Analysis of the physical and chemical properties of RGF members showed that the amino acid (aa) length differs greatly among plant species, with a minimum of 39 aa and a maximum of 277 aa in length. Relative molecular weight varies from 4384.86 to 30,049.9. Range of predicted pI values (isoelectric point) varies from 4.54 to 11.88. Most RGF proteins are alkaline (Table S2).

### 2.2. Gene Structure and Conserved Motif Analysis

In order to elucidate the evolution of the gene structure of this peptide family, we analyzed the intron/exon structures of *RGF* genes. The results showed that most of *RGF* genes generally do contain introns. Similar intron/exon gene structures were found in *M. polymorpha*, *S. moellendorffii*, *P. abies*, and in flowering plants, but the length and the number of introns are diverse (Figure S3).

Previous studies indicated that some CLAVATA3/ESR-related (CLE) and C-TERMINALLY ENCODED PEPTIDE (CEP) proteins contain multiple domains [17,18], but each of the RGFs identified in this study contains only one RGF domain. Many RGF domain sequences are shared by multiple proteins across species. There are 149 RGF domain sequences out of the 203 total RGF domains identified (Figure S4). Some RGFs share the same RGF domain. For example, an RGF domain sequence (DYAQPHRKPPIHN) was identified in six eudicot proteins (Solyc08g078840.2, Potri. 001G122600, Glyma.11G031700, Glyma.01G210300, MDP0000154697, and GSVIVG0100130600), while four monocot proteins (AC200065.5_FG009, AC202076.3_FG009, Seita.1G353400, and Sobic.004G333000) share the same RGF domain sequence (DYYGASVHEPRHH) (Figure S4, Table S3). These data suggest that some RGF domain sequences may be unique to eudicots or monocots during evolution. Our subsequently analysis of Brassicales also found that some RGF orthologous proteins have the same domain sequences as Arabidopsis RGFs, indicating the lineage-specific domain sequences emerged during RGF evolution.

Analysis of the RGF domains of different species indicated that, except for the conserved Asp at position 1, Tyr at position 2, and Pro in the second half of the RGF domain, the other amino acid residues differ greatly (Figure S5). Most RGF peptides were shown to be post-translationally modified by sulfation on the position 2 Tyr residue and hydroxylation on a Pro residue. The asp–tyr is a characteristic paired residues shared with other known sulphotyrosine peptide ligands such as phytosulfokine (PSK) and plant peptide containing sulfated tyrosine 1 (PSY1) [19,20]. These conserved sites are likely important for maintaining the primary function of the RGF family. Other amino acids may determine the function of the peptide and the binding specificity to the receptor.

In addition to the conserved RGF domain, RGF proteins contain some similar motifs, and these motifs may also play an important role for the function of RGFs. To further reveal the diversification and functional potentials of RGFs, ten distinct motifs were identified in these proteins using the MEME motif detection software (Figure S6). The results showed that most RGF members contain Motif 1 and Motif 2. Some motifs are unique to only some lineages, such as Motif 3, Motif 5, and Motif 9, which appeared only in angiosperms except for basal angiosperm *Amborella trichopoda*, suggesting that such motifs are formed after the differentiation of angiosperms. Motif 4 is only found in some monocots. Motif 7 is only found in some dicots. Motif 10 appears only in apple RGF members, indicating that the *RGF* family genes are evolutionarily diverse and are lineage-specific.

### 2.3. Phylogenetic Analysis Divides RGFs into Different Subfamilies

**Figure 2:**
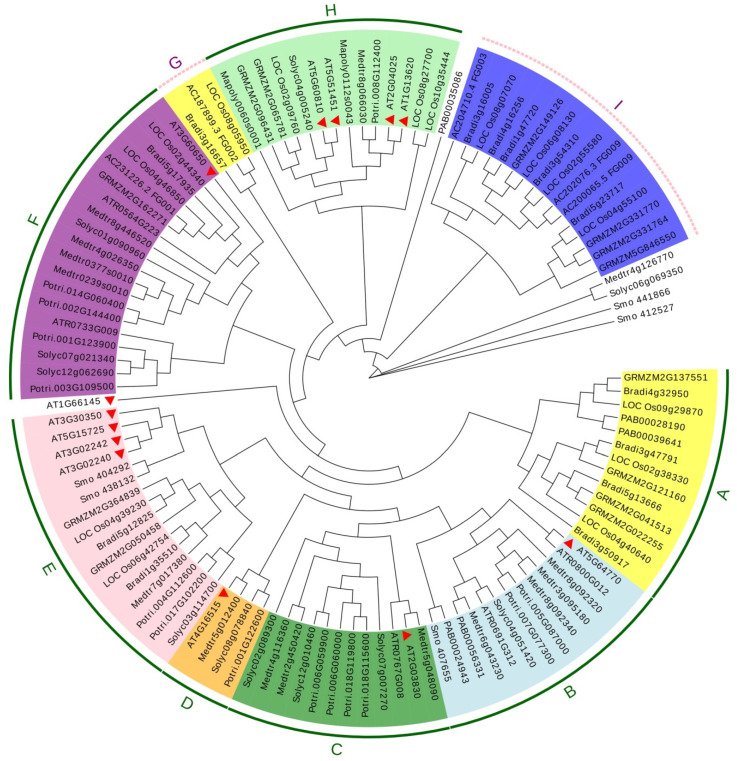
An unrooted approximately maximum likelihood phylogenetic tree of the RGF proteins from 11 out of 24 selected species. Full-length amino acid sequences of RGF proteins from liverwort (*Marchantia polymorpha*) and several seedless vascular plants (lycophyte *Selaginella moellendorffii*, gymnosperm (*Picea abies*), angiosperms (*Amborella trichopoda*, *Arabidopsis thaliana*, *Solanum lycopersicum*, *Populus trichocarpa*, *Medicargo truncatula*, *Brachypodium distachyon*, *Zea mays*, *Oryza sativa*)) were used to construct the phylogenetic tree. Members in the same sub-branch were marked by the same color. Arabidopsis RGF proteins were marked with red arrow heads.

To further explore the evolutionary relationships among the RGF family members in plant species, full-length amino acid sequences of RGF proteins from liverwort (*Marchantia polymorpha*) and several seedless vascular plants (lycophyte *Selaginella moellendorffii*, gymnosperm (*Picea abies*), angiosperms (*Amborella trichopoda*, *Arabidopsis thaliana*, *Solanum lycopersicum*, *Populus trichocarpa*, *Medicargo truncatula*, *Brachypodium distachyon*, *Zea mays*, *Oryza sativa*)) were aligned and inferred a maximum likelihood method phylogenetic tree. We showed that RGF family members could be divided into nine subgroups (from A to I) according to the phylogeny (Figure 2). Except for groups A, G, and I, the other groups have at least one corresponding ortholog from Arabidopsis. Interestingly, the distribution of AtRGFs is similar to the grouping of Arabidopsis RGFs, based on their gene expression patterns [16], suggesting the presence of coevolution of amino acids and expression pattern during paralogous divergence.

In group H, AtRGF1, AtRGF2, AtRGF3, and AtRGF5 are expressed primarily in the quiescent center (QC) and/or columella cells [15,16]. Although none of the single mutants, *rgf 1*, *rgf 2*, or *rgf 3*, show obvious root developmental defects, the *rgf 1 rgf 2 rgf 3* triple mutant exhibits a short root phenotype with a greatly reduced meristematic cortex cell number (REF). Expression of *AtRGF1* in *rgf 1-1 rgf 2-1 rgf 3-1* triple mutant can restore the reduced meristem size of the triple mutant [8], indicating the functional redundancy of these subgroup members. Moreover, there are fewer group H members in other species compared with Arabidopsis. For example, there is only one ortholog in poplar, medicargo, or tomato. We also constructed the phylogenetic tree for some Brassicaceae RGFs (Figure S7). The results showed that most of the Arabidopsis RGF members have corresponding Brassicaceae homologous genes, suggesting that group H members may be specifically expanded in certain lineages.

### 2.4. Pairwise Similarity Approaches Support the Existence of Distinct RGF Clades

**Figure 3:**
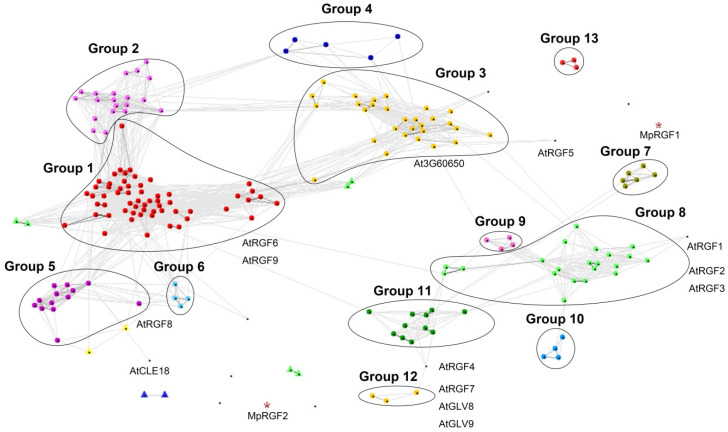
CLANS sequence similarity analysis of all 203 RGF proteins found in the 24 selected species in this study. A 2D clustering diagram is produced in CLANS based on their sequence similarity. Sequences are represented by colored dots. Same color dots belong to the same group. Gray lines connecting the dots correspond to BLASTP values better than 1.0 × 10^−4^. Characterized Arabidopsis RGF members (11 known AtRGFs, At3G60650, and AtCLE18) were labeled. Two putative RGF sequences from *Marchantia polymorpha* (MpRGF1 and MpRGF2) are also highlighted with two red stars but they do not cluster into any groups (Supplementary Data).

Since the RGF domain is too short and the whole amino acid sequence is highly variable among RGF members, the phylogenetic tree is with low statistical support by bootstrap. In order to confirm the accuracy of the phylogenetic tree and to further elucidate the evolutionary relationship of the RGF family members, we performed cluster analysis based on pairwise similarity by using CLANS [21]. We used full-length amino acid sequences in cluster analysis. The cluster analysis divides the 203 sequences into 13 groups, which are similar to the topology of the phylogenetic tree (Figure 3, Supplementary Data). Members in the same group share more sequence similarity compared with the proteins from different groups.

**Figure 4:**
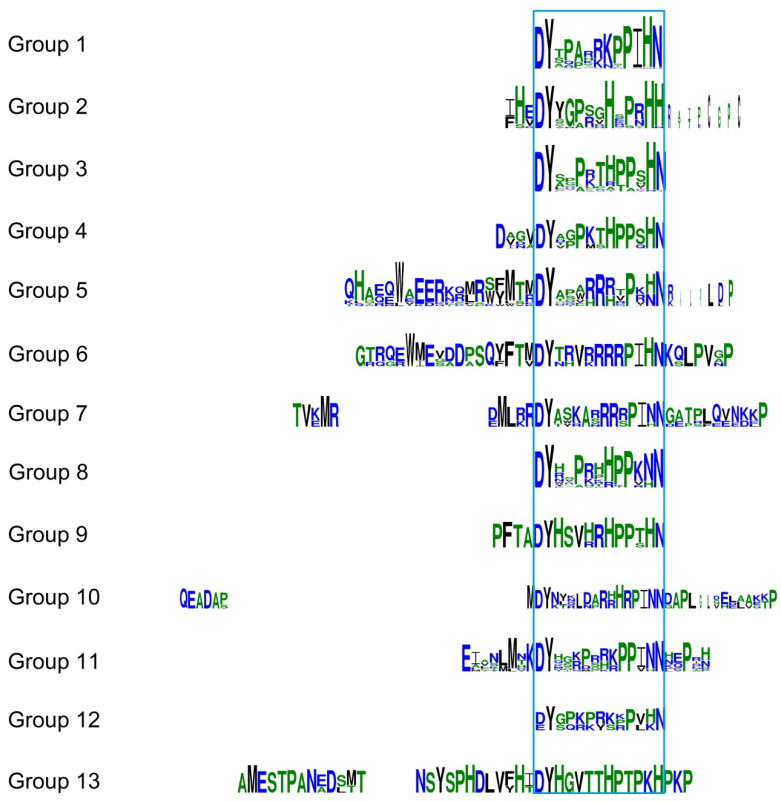
Logos of the RGF domain and adjacent conserved motif sequences in each group. The previously described main RGF domain is marked within a blue frame. Group specific residues are also marked in the various groups. The size of the letter indicates the degree of conservation of the amino acid in the group.

We aligned each cluster and built sequence logos to explore whether there are some sequence similarities within and outside the RGF domain. Besides two residues (Y and P), which are almost unchanged in the RGF domain, some protein sequence regions outside the RGF domain are also conserved in different groups (Figure 4). These relatively conserved specific residues are likely to play an important role, such as in the recognition signals for peptide cleavage, the transformation of precursor proteins/pre-propeptide, processing, transport/trafficking, functional diversity, and recognition of different receptors [22,23].

### 2.5. Evolutionary Patterns of RGF Gene Family in Plants

The expansion of gene family usually results from different types of duplications, including whole-genome duplication (WGD), segmental duplication, tandem duplication, and transposition events [24]. Gene duplication is an important driving force for evolution and provides a basis for the diversification of gene function. In order to better understand the evolutionary history of the *RGF* family genes, we analyzed the segmental duplication and tandem duplication of the *RGF* family genes in angiosperms. Our results showed that tandem duplications were not found in some monocots, while several pairs of tandem duplication gene pairs were found in Arabidopsis, poplar, medicargo, and soybean. Segmental duplication analysis through the PGDD database showed that *RGF* family genes, such as *AT2G04025* and *AT1G13620*, undergo frequent intra- or inter-species segmental duplications during evolution. The discovery within the region of the segmental repeat indicates that the pair of genes is likely to be repeated from the segmental duplication. Segmental duplication was observed in maize, rice, *Brachypodium*, soybean, poplar, tomato, medicargo, and some other species (Table S4). For example, several pairs of *RGF* genes in poplar have similar *K_s_* values (from 0.3835 to 0.4785), suggesting that the duplication events in this species occurred within the last 21.09–26.29 million years, indicating that these genes are duplicated in the recent large-scale genome duplication event of differentiation of Populus at around ~60 MYA. Therefore, tandem duplication and segmental duplication may be the main duplicated models leading to the expansion of the number of *RGF* family genes in angiosperms. In addition, the *K_a_*/*K_s_* ratios were calculated to estimate selection pressure among duplicated gene pairs. We showed that most of *K_a_*/*K_s_* ratios were less than 1, suggesting that purifying selection is strong for most *RGF* family genes after segmental duplications or WGDs (Table S4).

### 2.6. Selection Analysis of RGF Family Genes

Analysis of non-synonymous and synonymous substitution rates (ω = dN/dS) is usually an effective method for detecting positive or purifying selection on protein-coding genes [25]. To determine whether positive selection has an impact on the evolution of the *RGF* gene family, we calculated the evolutionary rate of paralogous gene pairs in the phylogenetic tree and found that most of the evolution rates were less than one (Table S5). We also analyzed the selection properties of the RGFs in different groups according to the phylogenetic tree. Site-specific model was used to investigate these groups and likelihood ratio tests were conducted in three pairs of models (Tables S6 and S7). Our results showed that although the ω values for each group were significantly different, most ω values were substantially lower than 1 (Table S6), suggesting that the RGF sequences within each of the groups are suffered from strong purifying selection that ensures their conservative functions among lineages during the evolutionary process.

### 2.7. Expression Patterns of RGF Genes in Different Tissues

To examine the divergence on expression patterns among RGF copies and to gain a broader understanding of the putative functions of RGFs in other plants, we analyzed the expression of some *RGF* genes by using publicly available datasets. A heatmap of 11 *SlRGF* genes of tomato and 18 *ZmRGF* genes of maize in different tissues and organs was established by MeV 4.9.0 (Figure S8). Most *RGF* genes were highly expressed in roots at different development stages, providing an important clue for their functions in root development. Analysis of the expression patterns also suggests that some *RGFs* from different plants clustered in the same subgroup showed similar expression patterns. For example, *RGF* genes in subgroup H are predominantly expressed in roots, while *RGF* genes in subgroup B are mainly expressed in aerial parts, such as stems, leaves, flowers, and fruits. *RGF* genes in subgroup C are expressed in most parts of plants. These data suggest a strong regulatory conservation within each RGF subgroups during plant evolution.

Nevertheless, some duplicated *RGF* genes exhibit different expression patterns. For example, two maize paralogous genes from the segmental duplication were completely inconsistent. AC200065.5_FG009 did not found to be expressed in root, and AC202076.3_FG009 is expressed in most tissues except for root hairs (Figure S8, Table S4). The results indicate that neofunctionalization might have occurred during the evolution of the *RGF* duplicated genes. Moreover, we analyzed the expression profiles for some *RGF* genes in other plants. For example, some rice *RGFs* are expressed in inflorescence and developing seeds. Some soybean and medicargo *RGFs* are expressed in roots, leaves, flowers, nodules, and seeds. Most of the current studies on RGF have been focused on roots, but RGF may play diverse roles in plant development.

### 2.8. Biological Functions of the Identified RGF Peptides from Liverwort, Eudicot and Monocot

To determine whether the predicted RGF peptides from different species share similar functions as those in Arabidopsis, we selected three species with different root structures to perform functional studies. The early diverging extant land plant lineages (bryophytes) lack vascular tissues and true roots. However, they collectively possess all key innovations of land plant evolution, a multicellular diploid sporophyte, a gametophytic shoot apical meristem (SAM) with an apical cell producing 3-dimensional tissues, a sporophytic SAM, and cell fate specializations providing morphological and physiological terrestrial adaptations [4]. Unlike the dicotyledonous plant Arabidopsis or tomato, which has a taproot system dominated by the primary root, root architecture is more complex in monocots, such as maize and rice, forming a fibrous root system which comprises many types of branched root [26].

**Figure 5:**
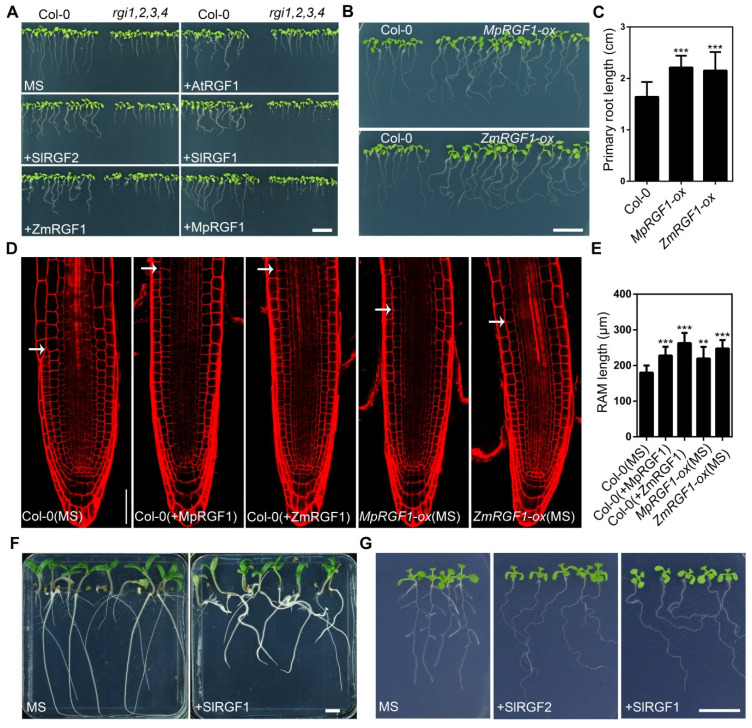
Functional analysis of RGF peptides or their corresponding coding genes from three different plant species, representing three kinds of root structures. (**A**) Six-day-old seedlings of wild-type plants and quadruple mutants grown vertically on 0.8% agar MS medium or MS medium containing 1.0 μM of synthetic peptide. (**B**) Seven-day-old seedlings of wild-type (on left) or plants overexpressing *MpRGF1* (up panel), or *ZmRGF1* (bottom panel) grown on MS medium. (**C**) Root length of seven-day-old seedlings as shown in (**B**). (**D**) Root tip from four-day-old wild-type plants and plants either overexpressing *RGF* genes or treated with RGF peptides. (**E**) Quantitative analysis of RAM in roots of four-day-old plants, as shown in (**D**). Error bars represent SD. Two and three asterisks indicate that the values are significantly different from the control at *p* < 0.01, and *p* < 0.001, respectively. *n* > 10. (**F**) Phenotype of 10-day-old tomato seedlings grown on MS medium supplemented with 1.0 μM synthetic SlRGF1 peptide. (**G**) Delayed lateral root development caused by the RGF peptide treatment shown in 12-day-old Arabidopsis plants. Scale bars are equal to 1 cm (**A**,**B**,**F**,**G**); and 50 μm (**D**).

We tested the functions of synthetic peptides corresponding to the RGF domain. We synthesized four 13-aa modified peptides (sulfated on tyrosine residues and hydroxylated on proline residues) derived from the corresponding RGF domains, MpRGF1 (Mapoly0060s0001, DYAEPDTHPPESN) from *Marchantia polymorpha*, SlRGF2 (Solyc07g007270, YSPAKRKPPIHN), SlRGF1 (Solyc04g005240, DYKSPRHHPPRHN) from *Solanum lycopersicum*, and ZmRGF1 (GRMZM2G065781, DYHSVHRHPPTHN) from *Zea mays*. Of which, SlRGF1, ZmRGF1, and AtRGF1 belong to the same subgroup. S1RGF2 and AtRGF8 cluster in the same sub-branch and share the same RGF domain sequence with two RGF members of soybean (Glyma.17G106100, Glyma.13G165000) due to the redundancy in the conserved domain. Figure S9 shows the sequence alignment of these proteins. Except for several key sites in the RGF domain, the amino acid variation in the other regions is very large (Figure S9). The results showed that all of the four peptides can trigger root waving in wild-type seedlings, similar to the function of the synthetic AtRGF1 (Figure 5A and Figure S10). Additionally, the wavy phenotype is more pronounced when seedlings were germinated on slanted plates (45°), indicating that the wavy phenotype caused by these RGFs was also independent of a thigmotropic disorder (Figure S11). The quadruple mutant *rgi1*,*2*,*3*,*4* showed reduced sensitivity to RGF peptides with no significant wavy phenotype (Figure 5A). Quadruple mutants also showed subtle wavy phenotype when treated with increased concentration of RGF peptides (Figure S12). Gravistimulation assays showed that these peptides affected the root gravitropic response (Figure S13). Among them, SlRGF1 and ZmRGF1, which belong to the branch same as the Arabidopsis AtRGF1, had the greatest effect on roots. Previous analysis indicated that *SlRGF1* is specifically expressed only in roots. Its effect on roots is consistent with its expression pattern. Similarly, the *ZmRGF1* is highly expressed in the root apical meristem of maize. The effects of MpRGF1 and SlRGF2 on roots, however, were relatively weaker. SlRGF2 and Arabidopsis AtRGF8 clustered in the same branch, they have similar expression patterns, and the expression level of *SlRGF2* in roots is lower than that in flowers, leaves, fruits, and other parts, suggesting that it may have a weaker effect on roots. Overexpression of these *RGF* genes showed similar phenotypes (Figure 5B). We measured root apical meristem (RAM) length in the primary roots of four-day-old seedlings (Figure 5D). Our results indicated that roots treated with modified synthetic RGF peptides or plants overexpressing *MpRGF1* and *ZmRGF1* showed a significantly enlarged RAM compared with their controls. Application of synthetic SlRGF1 on tomato seedlings altered gravitropic responses, indicating that this pathway may be more conservative in dicots (Figure 5F). Moreover, exogenous application of peptides also inhibits the development of the lateral roots of wild-type plants (Figure 5G).

### 2.9. Biological Function of RGIs (RGF Receptor Genes) from Liverwort, *Eudicot*, *and Monocot*

**Figure 6:**
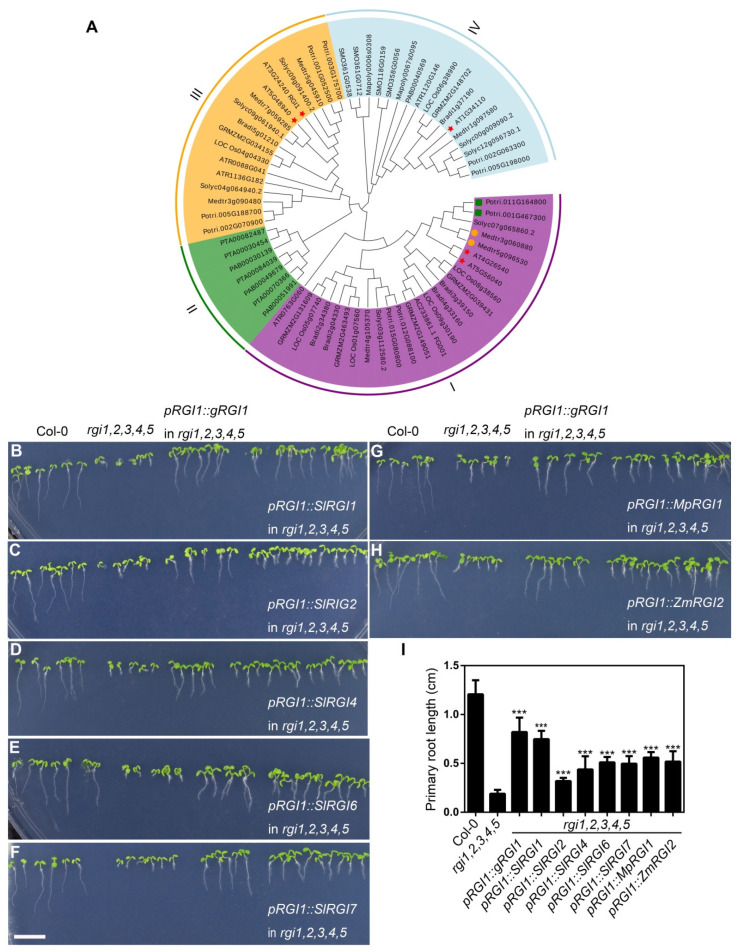
Phylogenetic and functional analysis of RGIs. (**A**) The phylogenetic tree was generated with MEGA 6 software using the neighbor-joining (NJ) method. Different groups are shown by colored branches. (**B**–**H**) Part of *RGIs* from three representative species can partly rescue the short root phenotype of the *rgi1*,*2*,*3*,*4*,*5* quintuple mutant. Eight-day-old seedlings cultured on MS medium for photographing; scale bar equals to 1 cm. (**I**) Measurements of the seedlings as shown in (**B**–**H**). Student’s *t*-tests were performed based on the differences between different transgenic lines and the *rgi1*,*2*,*3*,*4*,*5* quintuple mutant. *** means *p* < 0.001. *n* > 30.

The Auxin-TPST-RGFs/GLVs/CLELs-RGIs/RGFRs-PLTs pathway is known to be responsible for the maintenance of stem cell niche and the Arabidopsis quintuple mutant *rgi1*,*2*,*3*,*4*,*5* showed an extremely short root phenotype. To further understand the evolution and function of RGFs/GLVs/CLELs-RGIs/RGFRs signaling pathway, we analyzed all RGIs from selected 24 plant species. We identified 117 *RGIs* in 24 plant genomes. Both RGFs and their receptors RGIs were identified in the genome of liverwort *Marchantia polymorpha* but not in moss *Physcomitrella patens*, suggesting that the origin of ligands and its receptors may be simultaneous, and they may result from coevolution, which is in line with strong copy number correlation between ligands and receptors (Figure S2). All of the identified RGIs contain two important motifs (RxR and RxGG), which are important for the recognition of RGF peptides [14]. Phylogenetic analysis showed that 117 *RGI* genes could be divided into four major branches (Figure 6A). Group II contains only gymnosperm members. Most of the angiosperms are distributed in groups III and IV. The RGIs from *Amborella trichopoda* were early branching in each group among the angiosperm species, consistent with its phylogeny. The RGIs from monocots and eudicots plants are also well-separated in each group. It is worth noting that in eudicots, they undergo gene duplication after differentiation. Among monocots, maize, and rice form orthologous genes, indicating that they have differentiated in common ancestor. Expression pattern analysis indicated that the expression levels of most *RGI* genes in roots were generally high, which seems to be co-expressed with their ligands (Figure S14). In order to explore whether the related genes in the liverwort, tomato, and maize also have the similar biological functions with Arabidopsis RGIs, we cloned some of the *RGI* homologous genes from these three species and transformed them into *rgi1*,*2*,*3*,*4*,*5*. We found that most *RGI* genes from different plant species can partially restore the short root phenotype of *rgi 1*,*2*,*3*,*4*,*5* (Figure 6B–H and Figure S15), but none of them restored to the wild-type level. The degree of recovery was not as good as that of Arabidopsis *AtRGI1*, which might be due to the preference for codon bias. Overall, our results suggest that the homologues genes of *RGIs* in these species share similar biological functions in the plant root development.

## 3. Discussion

### 3.1. Origin and Expansion of the RGF Gene Family during Land Plant Radiation

Roots are an essential organ for plants to survive and thrive in land. They allow plants to anchor and acquire water and various nutrients in the soil [2,27]. Fossil evidence suggested that the roots were emerged during the Devonian Period about 416 to 360 million years ago (MYA) in non-vascular plants. Those roots were generally considered as hair-like organ rhizoids. True roots emerged in vascular plants is a multicellular organ with unique characteristics, including tropic response, endogenous branching, root hairs, root meristems, and root caps [5]. The latter two helped plant to perceive gravity. Clearly, the factors leading to the formation of both root meristems and root caps promoted the evolution of root functions and structures.

RGF peptides are recognized as critical signaling molecules mediating plant development, especially root pattern formation [8,9,10]. In this study, we performed a series of comprehensive analyses of *RGF* gene family across 24 plant species representing a variety of plant lineages. We did not identify RGFs and their receptors RGIs in algae. Bryophytes, including liverworts, mosses, and hornworts, usually possess rhizoids. They are extant representatives of early diverging land plant lineages. We identified RGFs and RGIs in liverworts, suggesting that RGFs may be originated following the initiation of plant roots. Previous studies showed that most plants had undergone multiple rounds of whole-genome duplication (WGD) events, for example, at least two rounds of tetraploidizations in Arabidopsis [28], and multiple WGDs in soybean (*Glycine max*) [29]. *RGF* genes have been expanded accordingly. Compared with most angiosperms, fewer RGFs were identified in bryophytes, lycophytes, and gymnosperms, which may be related to the limited diversification of gene families than angiosperms. It may also be due to the difference between non-vascular and vascular plants. Five *RGFs* were found in the genome of the basal angiosperm *A. trichopoda*. The tetraploid soybean genome contains the largest number of RGF proteins (28 RGFs) within the 24 species analyzed. Maize (*Zea mays*) has undergone multiple genome duplications, and it contains 18 RGFs. Other duplications might also be involved in the RGFs expansion. For example, several tandem duplications have been found in eight *RGF* genes from soybean and four *RGFs* from poplar but were not found in monocots in this study. While segmental duplications were found in angiosperms including eudicots and monocots, such as maize, rice, Arabidopsis, and tomato. It is speculated that multiple duplications occurred specifically in lineage, which may lead to the expansion of *RGF* genes.

The divergence of duplicated genes, such as diversified structures, expression patterns, and functions, provides an important genetic resource in plant evolution [30]. Most *RGFs* contain introns, but the length and number of introns are diverse (Figure S3). RGF domain sequences, especially four key amino acids residues, are all relatively conserved among plant species. This is important for the maintenance of their ancestral gene function. However, some motifs are lineage-specific, implying that functional diversification occurred among lineages. These identified motifs may also play an important role in the functional differentiation process, such as in the recognizing of signal peptides, protein folding, diversity of functions, and recognizing different receptors, which require future clarification via functional genetic assays.

Apart from gene structure, the gene expression patterns of *RGFs* exhibited diversification among different groups. For example, in group H, *RGFs* are expressed specifically in roots at higher levels. Whereas in group B, *RGFs* are expressed mainly in flowers, leaves, and shoots in Arabidopsis. Similar results have been shown in other plant species, such as rice, soybean, and Medicago. Thus, the duplicated *RGFs* diversified in gene structure and expression pattern that lead to neo-functionalization during the evolutionary process of plants [31], which may contribute to the formation of lineage-specific adaptive characteristics. Altogether, *RGF* genes of land plants likely originate de novo from a common ancestor of land plants. Lineage and/or species-specific expansions, and the subsequently occurring expression and sequence divergence also contribute to root gravitropic response and root structure diversity of land plants.

### 3.2. RGFs from Different Species May Have Similar Functions in Root Pattern Formation

Analyses including in vitro application of synthesized peptides and gene overexpression have been widely used to determine the functions of peptide related genes. Unlike the dicotyledonous model plant Arabidopsis, bryophytes lack vascular tissues and true roots, while monocotyledonous plants have a more complex fibrous root architecture. In addition, MpRGF1 may represent a relatively ancestral form. Functional analysis showed that the four of AtRGF orthologs from liverwort, tomato, and maize exhibited similar functions in regulating root pattern formation, indicating that RGFs have evolved conserved functions including meristem development and root gravitropism. Although their amino acid sequences share low identity, the conserved several key residues within the RGF domain may have determined their functional similarity. Since *rgi 1*,*2*,*3*,*4* and *rgi 1*,*2*,*3*,*4*,*5* mutants are not sensitive or show weaker sensitivity to high concentration of peptides, we speculate that in other species, the receptors of these peptides are likely to be AtRGI orthologs. In addition, SlRGF1 showed a stronger effect on root orientation and root length than SlRGF2. Expression analysis indicated that SlRGF2 is highly expressed in shoots. We propose that SlRGF2 may play an important role, mainly in tomato aerial parts.

### 3.3. The Co-Evolution of RGI and RGF May Contribute to the Root Development across Land Plants

In Arabidopsis, AtRGF1 can be sulfated by TPST and perceived by RGIs/RGFRs, a group of leucine-rich, repeat receptor-like kinases, thereby regulate root apical meristem development [12,13,14]. Therefore, it is essential that RGFs are perceived by RGI/RGFRs to function in root development. Here, we have also identified and functionally analyzed the RGI gene family in plants. Intriguingly, *RGF* genes and their receptors, *RGIs*, have been expanded in a coordinated manner. This is not unexpected given the close molecular interactions between these two families according to the gene dosage balance hypothesis, which suggests genes with strong interactions in a network are co-retained after duplications [32,33,34].

In this study, we only identified two LRR-RLKs which contain two essential motifs (RxR and RxGG) in liverwort *M. polymorpha* [14], suggesting the receptors, *RGIs*, are originated from duplications of LRR-RLKs, followed by obtaining novel functional motifs to perceive signals from *RGFs*. Hence, similar to *RGF* genes, *RGI* genes were found in liverwort *M. polymorpha* but missing in moss *P. patens*, suggesting that the emergency of ligands and their receptors may be simultaneous. The experiments with transgenic Arabidopsis provide unequivocal evidence that each of the RGI proteins from these three species is functional, since these *RGI* variants can complement the *rgi 1*,*2*,*3*,*4*,*5* mutant with respect to root length. Because expression of *RGI* coding sequences from different species led to a partial rescue of the short-root phenotype of Arabidopsis *rgi1*, *2*, *3*, *4*, *5*, our results demonstrated that the conserved functions of RGF and RGI maintain across the evolutionary distant species. Liverwort, tomato, and maize all have RGF peptides and their corresponding receptors. The RGF–RGI signal pathway may contribute to root development. The co-evolution of both RGFs and RGIs, regarding their functions and numbers, all promoted the development of plant root system. As the genome of Marchantia retains aspects of the ancestral land plants, we suspected that the peptide-signaling pathways might evolve with the origin of land plants. It is interesting that the Marchantia MpRGF1 and MpRGI1 proteins are functional even though they lack true roots, suggesting a pre-adaptation before the emergence of true roots.

Taken together, we analyzed the RGF family of land plants by comparative genomic and molecular biology methods, which provided an important theoretical basis for plant RGFs/GLVs/CLELs-RGIs signaling pathways, functioning as an ancient mechanism of root control/architecture in land plants, as well as the framework for further research on the RGF signal transduction process in plants.

## 4. Materials and Methods

### 4.1. Genome-Wide Identification of RGF Family Members

BLASTP and HMM-based search program HMMER3 (http://www.hmmer.org/) were used to identify RGF peptide-encoding genes. A profile hidden Markov model (HMM) was generated based on a multiple sequence alignment of 24 previously identified RGF sequences in Arabidopsis and rice [10]. The alignment revealed a region of high conservation of 13–16 amino acids at the C-terminus. The predicted protein sequences lacking the RGF domain were excluded. The proteins predicted from these species in this study were retrieved from Phytozome v12.1 [35] and Plant PLAZA [36] (Table S1).

### 4.2. Sequence Characterization

ExPASy (http://web.expasy.org/protparam/) was used to predict the relative molecular weight (MW) and isoelectric points (pI) of all RGF proteins [37]. The presence of signal peptides was predicted using the SignalP prediction program v4.1 (http://www.cbs.dtu.dk/services/SignalP/) [38]. The amino acid sequence is submitted in FASTA format with default settings. Gene structures of the candidate *RGF* genes were visualized with the Gene Structure Display Server (GSDS, http://gsds.cbi.pku.edu.cn/) [39] by comparing the coding sequences with the corresponding genomic DNA sequences. Conserved motifs in RGF sequences were identified by using the online tool MEME Suite v5.0.5 (http://meme-suite.org/tools/meme) [40] with the following parameters: number of repetitions = any; maximum number of motifs = 10; and with optimum motif widths constrained to between 6 and 200 residues. Sequence logo diagrams of RGF peptides were generated by using the online tool WebLogo3 (http://weblogo.threeplusone.com/) [41].

### 4.3. Phylogenetic Analysis and Pairwise Similarity Plots of RGF Peptides

The gene family trees were constructed with the full-length amino acid sequences of RGF and RGI members. MAFFT program [42] was used to generate the multiple sequence alignments of proteins. Approximate maximum likelihood phylogenetic trees were constructed using PhyML [43] with the JTT matrix-based model and SH-like branch support. Neighbor-joining trees were constructed using MEGA 6 software [44] with the following parameters: bootstrap values from 1000 replicates indicated at each node, poisson model, and pairwise deletion. The trees were annotated using EvolView tool [45,46].

For clustering analysis, full-length sequences of all RGF proteins were uploaded to the online CLANS server [21] which is a part of the MPI Bioinformatics Toolkit [47]. The output was used to create the 2D similarity plots and remove low-scoring BLAST searches with a *p*-value threshold of 1.0 × 10^−4^.

### 4.4. Analysis of RGF Gene Duplication

Information of the chromosomal location, length, and transcription direction of genes can be obtained from the PLAZA and Phytozome databases. Tandem duplication cluster was defined as a chromosome region containing genes within 200 kb [48]. The segmental duplicated genes were identified by searching the Plant Genome Duplication Database (PGDD, http://chibba.agtec.uga.edu/duplication/) [49]. To analyze the selective pressure acting on *RGF* genes, we estimated the nonsynonymous/synonymous rate ratio (ω = d_N_/d_S_) using a maximum likelihood method in PAML version 4.4 [50]. Seven site models (M0, M1, M2, M3, M4, M7, and M8) were performed for each group. *K_s_* value for each of the gene pairs was used to estimate the divergence time (T = *K_s_*/2λ), assuming a clock-like rate (λ) of synonymous substitution of 1.5 × 10^−8^ substitutions/synonymous site/year for Arabidopsis [51] and 9.1 × 10^−^^9^ for poplar [52].

### 4.5. Expression Analysis of RGF Genes

Expression data of tomato and maize *RGF* genes was collected from publicly available datasets from eFP Browser (http://bar.utoronto.ca/). Clustered heatmaps were generated with the MultiExperiment Viewer (MeV) v4.9.0 software [53], using Pearson correlation and the Average Linkage Clustering algorithm.

### 4.6. Plant Materials

The *M. polymorpha* L. *subsp*. *polymorpha* leaves used for gene cloning were obtained from Jin Li (Northeast Forestry University, China). Micro-Tom tomato (*S. lycopersicum* L.) seeds were provided by Juan Mao (Gansu Agricultural University, China). *Arabidopsis thaliana* ecotype Columbia (Col-0) was used throughout this study. Quadruple and quintuple mutants *rgi1*,*2*,*3*,*4* and *rgi1*,*2*,*3*,*4*,*5* (Col-0 background) were generated by our own lab [12]. Arabidopsis transgenic plants expressing different *RGFs* were obtained via floral dipping approach via transforming various constructs, which were generated by cloning the CDS sequences of corresponding *RGFs* into a *pBIB-BASTA-35S-FLAG-GWR* vector. The expression of *RGIs* from different species in Arabidopsis *rgi1*,*2*,*3*,*4*,*5* mutants were driven by promotor of *AtRGI1* for complementation.

### 4.7. Peptide Assay

Peptides were synthesized by Scilight-Peptide (Beijing, China) with a purity of 95%, and modified with tyrosine sulfation and proline hydroxylation. Seeds were sterilized using 75% ethanol (1 min), 1% sodium hypochlorite solution (10 min), and washed with distilled water five times. The sterilized seeds were then planted on Murashige and Skoog (MS) agar medium at pH 5.7. After kept at 4 °C for 3 days, plates were transferred to the chamber with 22 °C ambient temperature and 16 h light/8 h dark photoperiod. Seedlings were photographed with a digital camera. Root length was measured using ImageJ software.

For root confocal imaging analysis, seedlings were stained in propidium iodide (PI) dye solution (15 ng/μL) and visualized under a confocal microscope (Lecia TCS SP8). The size of the Arabidopsis apical meristem was analyzed by using the analytical functional components of Leica confocal microscope imaging software. The length of RAM region was measured from the QC to the end of meristem zone/the elongation zone of the root. The length of cortical cells in the RAM region was calculated by dividing the RAM length by the number of the cortical cells in the RAM.

## Data Availability

Data will be made available on request.

## Supplementary Materials

The following are available online at https://www.mdpi.com/article/10.3390/ijms222413372/s1.
