# Supplementary material for: Evolution of RGF/GLV/CLEL Peptide Hormones and Their Roles in Land Plant Growth and Regulation
Source: Int J Mol Sci. 2021 Dec 13;22(24):13372. doi: 10.3390/ijms222413372 (PMC8708909; doi:10.3390/ijms222413372)
Supplement: Supplementary file 1 [file ijms-22-13372-s001.zip › supp/Supplementary Data.pdf]

Group 1
>GRMZM2G331770 49
MKMRAPAVAFAALLMLLVVATRAHGIRLDRQLHDAINSSKEMADPRRTGDAEASSIAAGD
SVKEHCTRDGRCSGTKAEKALAHAEAAAEAKQQQGDSTGNDHRGTTVGGEAATQQGR
RHEEEAAAAPTDSYSSSSPEAPGVAARQRQTYPDIMDIAGMDYSPAARKPPIHN
>Sobic.006G237600 79
MKMRTPAVAFAALLMLLLLGTRAHGIRLDRQLHEAINSKKMADSKAGDAKASIAGDDSV
KKHCTPDGRCSGKVKKALAHPEAAAEAKQQKNNSTGSDHRSSTPVDGEATSQQGRRHE
AAAEATTPAASSSSPAPPGVPRQRQTYPDIMDIAGMDYSPATRKPPIHN
>Seita.7G260000 58
MRTCAVAFAALLLLLLLATRAHGIRLDRQLHEAINSKEMADPKSGDGEASIADDSVKKHC
TPDGRCSGAKVKKALAHAEATAEAKHQQVSSTGNGHTTTVDDAEAASQPRVARQRQQT
YPDLMDIAGMDYSPATRKPPIHN
>GRMZM2G331764 45
MTGLVRLPAGDRQGMICHMRLCVRALGSRTKAEKALAHAEAAAEAKQQQGDSTGNDH
RGTTVGGEAATQQVRRHEAEAAPAASYSSSSPAAPGVAARQRQTYPDIMDIAGMDYSPAA
RKPPIHN
>LOC_Os04g55100 34
MHTRTRTSLLALASLLLLLLATRAHGIRLDRQLHEAINNKEIMRDSKAEQSLNTARLMNK
HCTSDGHCNSGKVQRPVVQAEAGAAAKQQQQNQSLERSGDANQQEQETAPRQQEKTSS
TATATMTTYPDILDIAGMDYSPATRKPPIHN
>Bradi5g23717 92
MRTSSMVMASLLLFLLLATRAHGIRMDRQLHDALSSKEPAGDSKAAAQPSELAAHSASK
HCASDGRCSDAGKAKKAAAPAHAAKHHQPIPGGGKREEEEEEAAPSSRVLPRQENNAAA
TTYPDILDIAGMDYTPANRKPPIHN
>GRMZM5G846550 39
MKMRTPAVAFAALLLLLLQITTRAHGIRMDRQLHEAINSKKKMMADPKSGGGGEAASIA
AAGDSVRKHCAPDGRRRCSGTTKQMTPTTVVGSRYSEVAMIISAHAAPRFHEDYYGPSG
HEPNHH
>GSVIVG01012874001 194
MKTSLSICLLLLLLSLILSQVQGIRLDKGFSSAGHQKIQVEEGSPIKITSNGGLVGEVTLCRDG
HCSSGINRKLMTKTTSTSSTTPTTNSKNVKNGGKSGDQVPGREEENFYVNASPVSEHPEA
APEGYPDIIDIAGMDYSPARRKPPIHN
>Solyc07g007270.2 109
MKQVLLLSLLLLSTLVYEAQARNIPMLKENGDFGEVNIICKDNHCSSSGRNRKLMTKTTS
TSSPITTTTSTKNIKNEGNIKAHDTTILKGQSSSENFSINSSPETGHRKTSSDRHPDVLDLAG
MDYSPAKRKPPIHN
>Potri.006G060000 122
MRLSVVVSLLLLSFLLQQAQGIRLEKGFKQVGAQKVQAEKSPSKEKSNDGGVLGEAILCK
EGQCTGMNRKLIAVTTTTSSSTTITISKNEKNGENKADHISKGRSTNGEIVGENEKLSVNSS
PNSEHRVTKDHNEEIMDLTEMDYSPARRKPPIHN
>MDP0000164386 192
MTTSSLAVPLLLLLCLLLSQAQGIRPEKGFSSVRQQNKNIQEEENASMKRSNIAAGXAGEP
SNIASSGGTRKLISVTSPSSATTTSKNEKKGRKNRGDSEPKGENVNSHGGEDGNGKAKSP

VVSKQQEAAAAHQHYPDLADMTEMDYSPAKKKPPIHN
>MDP0000299658 187
MTTSSSAVSLLLLFSLLLSQAQGIRLEKGFISVRQQNNNIHKEENSVVKRSNIAAAGLAGEP
SNIASSGSTRKLISATSPSSTTTTTTSKNEKNGREKRGDPEPKGENIKSTTQHGGKNGNVRA
KSPVISKQREAAAAHQHYPDLADMTEMDYSPARRKPPIHN
>Glyma.13G165000 154
MKPSFVVSLLLLSLLLAKTQGIRLGKVSSAVQQQKQHDGESTLLKRSSTDTEEASLCKDN
ACTGNIKNRKLVNTPVSTAQSLSKNVEESKVDPSVNGNTRNVNTLSTSKHKDVPEEHYHD
LVEITEMDYSPAKRKPPIHN
>Glyma.17G106100 163
MKPSFVVSLLLLSLILAKTQGIRLGKVSSAVQQQKQHDGETTLLKRSNTGAEEASLCKENE
LCTGNIKNRKLVTIPVSATQSLSKNVEESKVDPSVNGNSRNVDTLSTSKHKDVPEEHYHDL
VEITEMDYSPAKRKPPIHN
>Medtr4g116360 134
MKPSCVVVSLLILSLLLTKTQGIRLGKGSLAFEQQKQHDEERSTMLKRSNTDSEEATLCND
QQKCTGNIKNRKLVTTSISTTKSLSKNVKNSEDVAHTSVNGNTRNVNLNGEAKEIKVKSL
STSNYLPEDLVDITEMDYSPARKKSPIHN
>Medtr2g450420 135
MRASFLISLVLLSLLLTKAQGIRLERGSLAARPNKHAKESNLLRRSNSGDEEAILCKDEQCT
GKIKNRKLFTTSTSAIHANSKKGSRDVAFNTNVKKVNAGGNEGNTTGNDKANGKEHEIR
VKQQQEEDVREELIEISDMDYSPAKRKTPIHN
>Potri.018G119800 125
MKMLTTPHPIIYPKSFSIYILAGMGRKLIAVTTSTSSTTITTSKNEKNEGNKAGSISKRRSSK
EEIVGEREKLLVNSWRNSDHKVNEDHYEEIMDLTQMDYSPARRKSPIHN
>Potri.018G115600 128
MKMLTTPHPIIYPKSFSIYILAGMGRKLIAVTTSTSSTTITTSKKENNEGNKAGPISKWRSSK
GEIVGEREKLLVNSWRNSDHKVNEDHYEEIMDLTQMDYSPARRKSPIHN
>Cpa.g.sc46.77 201
MISIDAGNNRKLSSTSATNLKNASNNPTIQKGLSENTEVKVNEDRQEDFPMNSPAPPSSSK
HQEEGYTDVMGITEMDYSPARRKPPIHN
>ATR0767G008 18
MRRAEILSLLFLCSMATAQGLSLNMELKRSLSRGIAVKKWVGFKERRRVAEEEIDVRKPVT
GNSRKLLNKISSIQLDFDLTSKVSDDSKRKELGNGGMERRSLSSMKKKGGVGEEYQKFSS
NDPLPDDLVDIAGMDYSPARRKTPIHN
>ATR0800G012 20
MASPQFLRVPLLLLLLILSEGRPLAVGNGVEERRRESVVGEELVPVNVHVDGYPDPMDIA
GMDYTPAGKKKPIHN
>Potri.007G077300 123
MAKISRNHLVLVAFFLLCFVSTCARAARTLREASNHGAEKKDQNDMFPSKENGLPDVEEL
VGMDYTPARKKPPIHN
>Potri.005G087000 124
MAKISRNHLVLVAFFLLCFVSTCARAARTLREVNNHEVEKKDHNNLFPSKENGLHDVDEL
AGMDYTPASKKPPIHN
>MDP0000180378 184

MAAIPCKHLVVVAFFLLCFVSITAQGRSLGLVSKRAGDAYQKNEDDQAVVLQDEINDLLA
MDYTPARKKPPIHN
>MDP0000144167 183
MAAIPCKNLVVVALFLLCFISITAQGRSLELGVVSKGAADAHQKKSHDLHDTTFKQKQDD
DQGVVLPDESNDLLAMDYTPARKKPPIHN
>Glyma.04G217800 162
MAVSPSKRFLLVAFLLLCFLSINATARILRETKGELAPVSTEKSHENQFMPNQGGEQDIGDL
ATMDYTPAKKNPPIHN
>Glyma.06G148100 170
MAMSPSKRCLLVAFLLLCFLSINATARKLRETKDELAPVLTTEKSHENQFKPNQGGGGEQ
DLGDLATMDYTPAKKNPPIHN
>Medtr8g092340 139
MAIAQNKRFLVVVLFLICFISIQARARTLKEKSNDVTSNTHNNKEDHMFKAKEEHNVGDE
VFSMDYTPARRKPPIHN
>Glyma.05G186400 153
MVVLNKRILLVAFFFLCFISIHARARVLKEKSNMVDSTSSDASTAHHSETHDHVFKPKEGK
YNANEVFSMDYTPARRKPPIHNLN
>Glyma.06G148200 169
MASSQIKRILFVAFIILCLFSSQTIAARTLKEKSNLTSGDANSVQHNKEAHAKVFKPKDEDV
NVGGDVFSMDYTPASRKPPIHN
>Glyma.04G217600 152
MASSQNKRILFVAFVILCLFSSPTIAARTLKEKSNLTSGDVNTVQHNKEAIAKVFKPKDED
VNVGGDVFSMDYTPASRKPPIHN
>Medtr3g095180 141
MVASQNKRILQVIFVLLCFFSIHIQVKARVLKEINNQSSEGVNKAQQHKEVHADMFKLKE
GGDNVGSDVFTMDYTPASRKPPIHN
>Glyma.08G144800 157
MSPNKRLLLVAFLLLCFITMTASARSLRDIKDDAVKKSTQTSFFKPNHEGVQDSNDELDTM
DYTPAKRNPPIHN
>Glyma.05G186300 176
MTIKRGQLGHVLCKCIVTHNTFIPKTLQELSKIMAMSPNKRLLLVAFLLLCFITMTASARSL
REIKDDAVKKSTQTSFFKPNHEGAEDSNDELDTMDYTPAKRNPPIHN
>GSVIVG01022266001 195
MAPCKCLILLALLLCFFFFFTTANATARSFGVMESNQYESAEKGHDSKFKPSEDGVLPEPE
EIGDLAEMDYTPPRKKPPIHN
>Medtr8g092320 143
MAVVPSKRLLIAFLLICFILTTTQARNLRDTNDGDEKGQSSLFKLNHEGTQTQEKTQTQEK
NDDLLDTMDYTPASKNPPIHN
>Cpa.g.sc72.52 198
MGRHSYNYNKGDLLLMVFILACFITATATARKLSNTWVDHEKRNHVEPRVEENIVDDVV
VMDYTPAEKKPPIHN
>Glyma.08G145000 149
MDSTSDARTYYQSETHDDHVFKPKEDKDNANEVFSMDYTPARRKPPIHNLN
>Glyma.16G164100 165

MLHNLSLEKLAGGKDMKLVLSTKVVLLIVFLVICYFSSSLIGGHARSVRTDPFGIHATHLG
QENLVNSEKQEKLKDDEGDLVSMDYTAPRRKPPIHN
>Glyma.02G079700 167
MKPVLSTKVVLLIAFLVICYFSSSLIGAYARSVRTDTFGIHATHLGQENLVNAEKKEKVIKD
DEGAQLVSMDYTAPRRKPPIHN
>Solyc04g051420.2 115
MAIVNQKTHWFLIMSFIFFFFLITCQARNLQVDNCELVQKNGNQQVVISTKEKSEYEDSVA
IDIDYTPARKKPPIHN
>PAB00024943 8
MGYKFMAAFLGLLLLACSSIQAAYTNTEEGNIHAENTLPLEYPVASRVDELLGQSSGENN
EIVSRKLLRGSVGRGLTESEKAEHSRFQAKRSESDDSLHVPDRDLASKNREGIANDHHELL
LSGSLASTRTPVSAIKVVNTGSGQHQPNLQITGKEVRVASLKSFLSSLYHNLHKRISLREVL
KKQILSDTMANNPGVSSNKHIPTSPVSLQVSQPFSDPYATSESPTPDDTTDITDMDYTPAQK
KPPIHNKSAP
>PTA00026785 15
VFLHLGIIILNMAYKFMAALLGLLLVASSSIHAAYTNTKESNTHAENTLPLDNMAASRVDE
LIGQSSGEHNEMVSRKLLRGTVGKALTESEKAKHGRLQTERSESDDSDLHVPNRNLASKN
RAGIANDHRELLSGSPASMRIPISATQVANTGSGHHLPNLQITGKEGRLTSLKSLLSNLDHN
LHKRISLQEVVKKQIVSDTMANNPGVGSNKHNPTSTVSLQVSQPFSDPHATSESPTPDDTT
DITDMDYTPAQKKPPIHNKSAP
>Medtr6g043230 145
MKQGQSRKIVLLIAFLVLCCFSSFLLGAYARNLRIDSFEFHARHTGNGGVKHEDYVVSTM
DYAAVKRKPPIHN
>PTA00022754 14
MILRSSSVSKAVISCKAVMTVTEFLALLIICSALASTSLQARAHHEGTGLVGRRTNFFTVKQ
SASHTQNQSDSSVENNNGMELASDRSIPKRLRRMESTQADRKLTNKDRITEKKPIQTRTG
AANGNVLRKPEAVKGAAHSSLASKSKRLRPSSGIQTTLSSLHESKVVSKEKMEGLAFQEL
EITTKRTLKSTASSPSQGQGKEEISNETNTYKPAPPSATSGRRGQTSPIPQSQTFHVSGSMTY
ESSEASDLSDIIGMDYGRARRKPPIHNKVPEP
>PAB00039641 11
MIIRRGSVSTVIIACKTIMTVTQLLALLITCSALASTSLQAQEHHEGTRLVVMQTNFLPVEQ
SASRTQYQSDSSVENNNGTELASYRSLPRRLRGMGWTLADGKLTSEERRTEKKPVETRTH
GAKGHVHRKHGVVTGAAVSSLASESKSLRPSSGIQSNLSYPQESKVISEEKMDGLAIQELA
ITTKRSLKSIASSPSDGQEKEEISSESNSYKPAPPSATSGHRAQASPTPEAQTFHVSGSMTSE
SNDASDLSDIIGMDYGRARRNPPIHNKAPKP
>AT5G64770 104
MAIRVSHKSFLVALLLILFISSPTQARSLREVVRNRTLLVVEKSQESRKIRHEGGGSDVDGL
MDMDYNSANKKRPIHNR
>PAB00056331 9
MPNEAAQLRKWNLQPAFFGRRDGGVSQDSSVLVGYYKLEANAKLRGLNDIERRFIERLG
EENTAGMPEKHSSLKRILSIVSGSRPSMKMGYQNKFSGTQEAKSSSRTLCNIPETSKARPM
DEGAAMIPPKPQGDKDDGDVVNAMDYAPAHKKPPIHN
>PTA00019540 13
MMITTCKIVVIMLFALFFLSSNCRQMAIEAAQLRTWTLQPDFSGSRAHGVSQDSSVLVGY

DNLEAANAKTRGVNDIERKYAEHQGEENTAGTPEEHRSLKRSLLLVSEARPSMKLGSQNK
FSGTQEAISCSRTLCNIPETSKARPIEEAAAMTPPQPEGDQDVGDLINAMDYAPAHNKPPIH
N
>PAB00028190 12
MTEYQQQNEQTIKELLSCYHVEEEPPDEDDPHNIQIIDIEEEREVEDPRRPETASAKELENL
RGDGKGTRKHSDRIYDYDVYNDLGNPDKSKDLARPVIGGEDIPYPRRCRTGRSPTETDPL
AESRVEEPTPLYVPRDETFEEIKQDTFAAGHEKIDGLAIQELAITTKRSLKSIASSPSDGQEK
EEISSESNSYKLAPPSATSGHRAQASPTPEAQTFHVFGSMTSESNDASDLSDIIGMDYGRAR
RNPHIHNKAPKP
>AT4G16515 101
MSCSLRSGLVIVFCFILLLLSSNVGCASAARRLRSHKHHHHKVASLDVFNGGERRRALGG
VETGEEVVVMDYPQPHRKPPIHNEKS
>Smo_407655 4
MVNAASTALALGCCCCLLAAACLLCSADASRATVEPHSLRPPVGAKKQASRRMLTEGAR
GQARRVLKALDFEQAPPPAQGPLGYSSSFTSKSHIPSDSDELMSMDYTPVHRKPPINNRIPY
LPVHYSPGRGQ
>Potri.001G122600 120
MSTLFLLLLICLSLHACNARFLRLTDKETKKQVLNKVLVQDVDKLNINRTTLAPSEMMPP
VSEEQGAQGQEVVGRDETITRKISLLSATTMKKKDEEAFMKQEEGTKGATSGNKVIVSSQ
ADLQQSSKIIIEGLKGRGRSMLGSTANDTEEAVRSEENDIAEDVAVMDYAQPHRKPPIHNK
KP
>Solyc08g078840.2 110
MFINMSKFLVYLLVCICISLHACSARPLASMDDKENKILLSSKDVKFLTSDISKTEGRIIMSE
DIGKDDGKLMWKKRSIVARKQLDDEEDKGNKVKISFHEGAQVKISRLMLESPPSTQHEEE
AVNSIEKEPVEDVVVMDYAQPHRKPPIHNTKH
>GSVIVG01001306001 193
MSSLLLLLLLSLSMHACNARHLGVIGKENREVHLLSKGWVQVVEEADQVHKISLPPRLIP
SMETAEKLQPQQILPVGSDKRNPQENWGGVKNMIEIGSEALSKEEEDSKRTTTGSKIIVLSS
HEDLQQATKATKIEGWKRHSRLMLGSAPHDVEETVDSKEGDIVEDIVVMDYAQPHRKPPI
HNKDP
>Glyma.01G210300 150
MHARPVLFSLSLTIKDTTHKELDKFKLLETLTATSSIVKNYKLEANQPQQQKVVNNEINTC
ESCSSVSSTLKETIVEAASVKIIPSDSGEHAAGTSFQMDSPSFLASQDERRHARSMLGPAQH
NDEETVVTNASDTEEDIVEMDYAQPHRKPPIHNEKP
>Glyma.11G031700 166
MNSLSLSLAMLLLLLCVSVHACSARSLYPSLTKKDTPHKELDKLKLLEALTASSTVKNYK
LEENQPQQQKVANNEINTYESCSGASSTLKETIVEAASVNIIPSDGGEHATGTSFQMDSPSF
LATQDERRHARSMLGPAEHNDEETVVTKTSDTEEDIVEMDYAQPHRKPPIHNEKP
>Medtr5g012400 147
MASLPLLLLLILCGSLHACTPRPFTEKDTTKYHFNKELNNVKLLETLTTSSVKDNKLEENP
ALQQKVTNDEINICVSCSSASTLKKVIVGSTFQTGTPSLLAAKDERGHARSMLGPATHHVE
ETMVINTNENAEDIVEDYAQPHRKAPIHNEKP
>MDP0000154697 182
MEYMPHRVHTKYGRWWGKVDLSMSTKELHVTVLPCSFASFIPMSLPPTPLMSWVLFNLF

FGNXRGLNLRHGARATLLSVIVVEGHLQLLIVIIRVNHASSKIEVHLRFLPTESGYRAYHSV
EDVGNVLRRSETSMSSTRPSISVEFQSKEVEVKDAETVPTLESXPNDDGSRKQGHVNFGP
TSAQDIFTFSQIHQKKSIKSEEIEYTIQGLERQTRTLLGSATLHDQMEEDKDPKGNEATEDV
GVMDYAQPHRKPPIHNEKS

Group 2
>Seita.4G052800 60
MSTPITGWGQSCVPEQRCIYIISGSNGSSPSNKDDSSSVFIIPSKLHYRTVPISRSLHRDQMK
TSLPLVAAVIVLLLVMARVEGIRLDSESHEAFSNQMVHKSGEMAVKNTDNEPSGEKMEESI
SEEKDRVGHRLPEIHVDYYGPRGHKSRHH
>Sobic.010G062400 76
MMKNSLSLVMVVIVLFLVTARVQGIRLDAESHEAFNKQMVDKPGVMGVRDTDNEPSGG
KMEESISEEKGRVGHSLPEIHVDYYGPRGHRPTHH
>GRMZM2G149126 44
MKKSSSPLVAVAVVIVLFLVMARVQGIRLDAESHEAFSNQMVRKPGEMGATYADSESPGE
KMEGSISEEKDRAGHGLPEIHVDYYGPRGHKSRHH
>LOC_Os06g08130 24
MHKQIYATLYIPSETWMQLSRSRYYTSIADMSAAITRWDHGFASEANDKIEMKTSLPLVAA
VLALFLMAATVQSIRLDAESHSAFSKQTVNNTSGDNVVAVVVAKTDGEPSGEMEKAISEE
KSRVGHELPEIHVDYYGPRGHNPRHH
>Bradi1g47720 87
MVKTCSPLAAAALALLLMFATARAIRLDAETNAALSNQLLNKSVDKGVMIKADGVEPSS
SSGEAEETISEEKDRTGHRMPEIHVDYYGPRGHNPRHH
>Bradi4g16256 94
MKISSPLVAAAMVLLLTVATVQGIRLDAEIHAALSNQELNLQRSGDEGAVSSLDAVSEEKE
RARHREPENDIHVDYYGPRGHIPSHN
>LOC_Os02g55580 25
MRMSKLSMATAVILLLVVMAMEVEGIRLDAETRAATSNQMVNKSTENVPKDSGDSLGEA
KRSIAGNEVRAVAHKLPEFHEDYYGPSDHTPRHH
>Seita.1G353400 63
MRTSVSLAAAVFLFLLLTAMEAEAIRLDAESRAAVSQSQQQTVNKSAENLVQKQEVAPGK
SSVGESETKRSIAGQEVVRATTHKLPEFHEDYYGASVHEPRHH
>Bradi3g54310 90
MRSSSYLPLAAAAIFLLVLSRAMDQARAIRLDAETRASVSGSQTAIDKPIDNVVDKDSITTS
VGDTKRTTSAAVAAEVRAVAHKLPEFHEDYYGPSDHSPRHH
>Sobic.004G333000 67
MRTTSTSLSLAAAATVFLLLLLTTAMEVEAIRLDAESRAAVSQQQQVNVANQKPSETLTTE
KNAPGGERSSPAGESETKRSVAVAVSVAGQEVVKATAHKLPEFHEDYYGASVHEPRHH
>AC200065.5_FG009 43
MQMRRTATASLPLATAAVFLLVFLAATTVEVEAIRLLDAESRAAANRQQAIVPSASVLVTT
QQQQQQHGRRQGETGLTRTVDAPASREERSSSSPAPAAAAAGENSEAGRSVVAVAVAGQ
QDQAVEAAAHRLPEFHEDYYGASVHEPRHH
>AC202076.3_FG009 52
MRTTTSLPSAAAAATVLMLLLLTAAVEVEAIRPLEAVKPQQQANVAINVVSGPPAAFSSPA

KRSVSVDGQEVVKATLTAHKLPGFHEDYYGASVHEPRHH
>Solyc12g010460.1 117
MKQVILLIFLLYLSTLIHEAQGRHLKKGHKLQEKTNLIGSSKGVIGEVSTLCKDGHCSSFTT
KGRLTKVAKKSHHWLPNIHEDYYGPRGHRPRHH
>Glyma.09G094100 171
MKPYSLVISLLLLSLFLTKAQGIRLEKGSFAAQQHKQHDQEESNLLKKTNNGANKEAIVC
KDEQYCTGTLKKRASRVSRKPKNQLPHIHEDYSGPRHHRPRHH
>Potri.006G059900 126
MRASLVVSLLLMLFLLQEAQGIRLEKGFMQVGAQKVQADQDNTSLKQISTGVLGEEVILC
KEGHCTSTLKKRLSRSVSKKSSHHWSPRIHEDYCGPRHHKPRHH
>LOC_Os08g07070 31
MKSSMVLASVILLIMAATAHGLRLDMGLHAALNNEGMLNSKWQSTANRPIDTRRASNDR
RGPGRSRTRPPKMSNPHDMDPRFSEDYSGPGGHSPNHHRTTPCGPC
>Bradi3g16005 86
MRISMAPASVLLLLVLLMAAMARGLRLEMQLRATLGKEPASEWRASPGSRPMNKPRASN
GGHADLGHKEEAIGATAALDAVKDTEVKRMKKMGSYPRFSEDYSGPSGHSPNHHRATPC
GPC
>Sobic.007G055400 69
MLSSMVFGSLILLLATRAYGLGRLDMQRHTAQHQQLHKKVDSQKEEIQIPADFTWQRQPP
SSPSGRRPAAGVRDDASDSRNGLGGGSTTPEHRLTTSTGRAAEKDGEAKKKKTTHRGPH
GVVAPRLIHEDYVGPSGHSPNHHRAIPCGPCKH
>AC204710.4_FG003 37
MAFGSLLLLFLLATRAYGLGRQDMQLHTAHLPMNTKVNSQKQEIPADFKWQLRPPAGTL
DSRRNGLGGSTTPPEHRRLATTTGRAAAAKEEDGGGEVKKTMWRTHRREVAAPRFIHQD
YAGPSGHSPNHHRAIPCGPC
>Seita.6G049000 54
MRCSMLFGYLIIIILLARAHGRLDMQLLSALGKEEMPSALKWQRPPQPSSSAGGRSADTV
SRATERHLMITAGAAAEEDGEVKKMRAHREEMAVRLIHQDYTRPSGHSPNHHRTIRCGPC

Group 3
>Glyma.01G136600 164
MSSTAYLLFFLCISLHACYARHLSPPNKKMEEKSHFSIKSDEKNGLDSSPKQLNEGHNKTE
TRLVGDSVKPRNRRSTNHHRVHKATENASGSSLQKSLVSVSWRVPHNNNKKPRQKHSGF
DLDYSPPKTHPPHHN
>Glyma.03G031600 160
MSSTAYLLLLFLGISLHACYARHLSPLNKKLEEKSYFSIKSDEKNGLDSSPKQLNEAGHNK
TETRLVGDSVKPRNQRSTNHHRVVHKATDANASGGGALQKSLVSVSWRVPHNNKKPRQ
KHPGFDLDYSPPKTHPPHHN
>Medtr4g026350 138
MSSSSYILLLFLCIISLHACNARRHPSSDLDKKMEKKPHFSLKINVEKNAFDSSRKKLSEGD
NKMKAEFVADSEKSKHQRSTNQKVLKDMRKVSAELQTKSHVSVSSLRVPHNKKKHSEK
HPGFNLDYSPPKTHPPSHN
>Cpa.g.sc49.161 202
MSITSWLLLFLLFLSLHSCNARPLHNKLQNKHHSSIKNQEKKAFEEISAVELAKMRSSLSK

QQETTKGEEIIETETQRITKARIAKTPKKIQGEKTSDNHQTESLVSVPWRVPHDKNRRREK
NPGFNLDYSPPKTHPPSHN
>Potri.014G060400 121
MSVITYSCYFLLSILCISLHACNARPLADIDKKPEKKFQIISNQTSDQKEISVTIVSKADSSSS
NEFGAAKEDSIAKTDQLDDNAQKLKDSRAKQKSMSDEKVKDSGAALKESLVSVSWPVP
QKKRGETHPGFNLDYSPPKTHPPSHN
>Potri.002G144400 127
MTPMRAALAFLNLSIPLADINNNPEKKFHIIPYQNDQKEISVTTVPTVESSSSNEFGAAKKD
SIAKTIGDNIAQKLKDSKAKQKLDSVDEIEKNPGAVKKESLVSVSWRVPQKKRGERHPGF
NLDYSPPKTHPPSHN
>Glyma.01G079000 175
MSFTYHLFFICIFLHACSARCFSTLHNKLEKKPHFSIKGDEINGVESFCKHLAVANEVNYKI
KPRKAKTTNQKVLKAIRKIQKPSKKDPGFNLDYSLPKTHPPSHI
>Medtr0377s0010 136
MSLRMSVTYLLLFLCICFHAYCTQDMKLEKTPHFSIKADNINDFESIPNHFLMMNEGRKM
KTWLVNTQKSKKELCTNQKVFKAMRKDSSEINKLSEKNPELDLDYSAPRTHPPHHN
>Medtr0239s0010 140
MSLRMSVTYLLFFLCIFFHAYCTQDMKLEKTPHFSIKTDNINDFESIPNHFLMMNEGRKM
KTWLVNTQKSKKELCTNQKVFNAMRKDSSEINKLSEKNPELDLDYSVPWSHPPYHN
>Spipo1G0079900 81
MLPGSKDAIEKNTTTMQLQPTTENLSRLPRPADGGAAIRTEDGGPAAIEENLKAPVKASAV
ARSQSHGAVSWRVPRDAQRGHPGFNVDYDAPKTHPPSHN
>ATR0564G223 16
MANQRRNLTISPLLLILFLSLIVHTCSARLLLAASHAERVHHHSSKGREGESVRIHSNTIMK
LLRKEDQGSRRKMGKSLSVVQNRSLTAVSRRSSRGKHGPGFHLDYSPPLTHPPIHN
>Glyma.08G232200 158
MYLPSSLFIIMFFFLSLNVCTARPLVVAVEEASNTTQINISRKVSENVKKQPLELNGMTSEGI
NAKINNMGAEREESSEHTMRKVHPNKALGFVPVKPLAVLVSWHVPHSKHDQNPGFHSDY
ARPRTRPPSHN
>AT3G60650 108
MNTNSYSMAQDNEEITKLEAPSTNKTNTLLSQSHAVVNHGDNGQINGKKTKEIHRVKRA
SDKKVSSKRVSRTWKIPKYPKKQPKSDQEHPGFNLDYMQPTTHPPHHN
>Potri.003G109500 132
MPIKSFLLSIFLLSLSMHALCNARNIGFVKEGFHAEVYFSAKVGEKVEHSMDSVHGTNDR
EDISEKSTPGAAIITQESKDSKAITKSSGHDQNPIKPLVPVSWRVPHKNLHQIPGFQSDYAM
PRTRTPSHN
>Cpa.g.sc3.318 200
MHSTISTLFVFLFLLSFFHACAARHFRPSEDSSTCSKGFENVNHHQQKIDPLISEEDDDGQE
GTDAGAMMQEEADKETSSSEFINGVKKKMSNKGEISSGVSWHVPRNNHHNELIPGFFSD
YSNPRTRPPSHN
>Medtr8g446520 142
MYLQLCIFILLLSLNVCTSRTLLGVKDTQINISEKESENVEKLPLKSNGATKTDIKVRNNNI
GVVQVHHIKQEISDHDPHRKEGKNPGFYSDYSRPRTRPPSHN
>Solyc01g090960.1 111

MSITKITTLLMMIFLLFHACNARSLGIMNKNEEKPNVARVPSTPSDVKAKHSPSKPNKSVK
ENDEKDHHYGVSVSWKVHQQKKEDPPPEFNLDYLPARTHPPVHN
>LOC_Os04g46850 32
MQLKLTAAVAALLVVVFFALFVGSCEARRLRALGRVSSLKPSSSPPTPYKDAASMKLHGS
DPTNQKKDLSSTSMDHHMASGDDAKAKDGVAMASPGAVQTTIVVRVSNRLSHQERRVD
TAFHLDYAGPRTHPPSHN
>GRMZM2G162271 51
MPSGLNSPLLLAPKGQLMKQNTAVLLLSIVLLATSCEARGLPVHGKVVRSSASQSHRLPAG
KDVAATSLTADGWLSTRQMEARHMTAQQADAKVKEGVKMAAASAAAGAVRATLPTPAV
TAVSQRLPRREDTGFHLDYAGPRTHTPSHN
>AC231226.2_FG001 47
MHSGLSSPLAHKGQLMKHSAAVLPVFLLSIALLASSCEARGLPVHGKVRSSSKSHRLPLPS
KDAAGASLKADGRLTRQMKARHTAQPEHQAGATAKEGMKMAAASAGAVRAAPTPSAVT
VSHRLSRPEDTGFHLDYAGPRTHTPSHN
>Sobic.006G172800 70
MPTGLSSALLAPKGQLMKHSTAVILPVLLLPIVVVLLASSCEGRGLPVHGKVRSSSKSHRL
PGKDVAATSLKADGWLSRQLMEARHMMAAQQADAEVKEGMKMAAASAGAVRATATPT
PADTVWQRLSQREDTGFHLDYAGPRTHTPSHN
>Seita.7G193200 57
MPSLSSPFAPKGRLMTSKHAAAAFLLILLSIVLFASPCEARGLRVHGKRRSSSKSSHLPAGK
VGIWLLMRKWLVLNVAATSLKADGWGTRETQARSTVHTAMDDDMMAQPDAKAKVGV
AMASSTSAAGTVGVTPVVRALSQREDTGFHLDYAGPRTHTPSHN
>Bradi5g17935 95
MPTFSSPLPPKTRQLKVTAATPTTVLITLVFLALFVRSCEPRHLLPHGKDSSSRSPPPAPCKA
DGSPINQKKNLSNAVDATGTLVDQVDAKVKKGVPMAMASSGAGVQTTIASRVSRRLSQR
ELQGEGTEFHLDYAGPITHPPSHN
>LOC_Os02g44340 35
MPSQSSPQTLNGKLKLNTSTTILLFIVFLFTLCIISCEARHDHLRISDKYSSKKSSLVPKDVA
GDDVVGSKQPIDQSVGKEVTLNAKMELAASSGGVRNTGPAVRVSQQLLRHRKHEDDQGI
HLDYAQPKTHTPCHN
>Potri.001G123900 129
MHSFKSSLFIFLLSLSMHAICNARNIGVVNEGFHAKVLFSAKVGEKVEHNTDTVHGTNDR
EGAIRGKSSAPGAAIMTRESKDSKAITKSSGIQILKSLNGLVVSLKAVNIEGYSTRRALSVA
GFGSNNVKKAMESGENEVEEDAEVIDYEPPHRTPPIHNRKTLVIAV
>Seita.1G262800 59
MPFSLTYPCILRDHYKQPHHSSMFSPSSTLALKGQLKLSTTTTVLIAAFILTLYINSCEARHL
RVHGKDYSRMLPASSSPPKGVTDGGASKGKMRSSSEVPVGSNMDTSMGNEAVIAAKKE
MASSGDSCRGSLRKRSEGMKVRSSLRERSVLGAESNSELIVGSNTTTAYTAETLVAMDYL
DAHPAPAVHNR
>Sobic.004G291300 78
MFSVSSPKGRLKLSITTTDLLVVFILTLYVTDCEARRDLRVHGNKHCSSKLTSSPPKGVIDG
AAGKGNVRSSSEIPVGSKVDASMLVNEGVTTAAKKEVASSGDSCRGSLSKAVEAIKVRPSI
GERSLLGADGSNSELVGSNMTAAYTAETLVAMDYLDAHPAPAVHNR
>Bradi3g50917 85

MFCLSSPPLALKGQLKLTTTTTSTILLVFFLFTLYITSCEARHLRANSKGSSNKYTLTSKDKA
AEVGDTEKQIRTASMVHGTGKTGVIAGSTIDQSMSTGATLINGSGNSSRGSNGQQRGSKA
TTAYTAGTLVAMDYPVARAAPAVHNR
>PAB00035086 10
MILTSSRSLEFLRSMILYKVVMAILILGLSFLPCKSSKLNGEDSVIMFQLHRQGSSVHSLVR
APGGDDTIRPQRVPVIKGQFPTSFTGKRRMLGENFAEKNVREDEKSLSRAHNRFNLEGSG
HENKVERPDHHHHHRRQHRRQAGHEAIFFRSKKRHLQVSGSTTVIVHEESSRTEWNSLA
AKKTNTVAWHKPGEKYDEPTFHVDYSGPKTHPPKNN

Group 4
>AC187899.3_FG002 46
MLLAYVLVFLLRLSASTCHATSRNQHLLFAGRAGDSTAPVHHRHTTKVLSDYNDVHYHP
AMMPSPASVELAGGIGGSPPPSLSAVEPSGVEGAPPPPPGKRDTSAPAMEPSPSPHSEGGAS
SSGDDDASATEQEDSAGYDLDDANVDYVGPKTHPPSHN
>Sobic.007G044000 75
MLLAYVLIFLLQLSASTCCYATSRNQHLLFAGRATALLPPAAAIHHHHHHRHTAKQAAEAR
AHAIVVDMQVLSDYNDVRRHYHPAMMARWGVGAPPRPPSTSPSVELLAGVGGSSSPPPP
PSLSAVEPNAVEGAPPPPPPLMPPAGKQETSAAMEPSPSLPHGEGASSSDASEMGHEESPSG
DDVDVDADYDGPMTHPPSHN
>Seita.6G058000 62
MLLAYYLLLFLLHLSASTCDATSRNQHLFLPSPAIHDHRHAANKRGIYPCMQDLSYYDAH
YYPAMMARRGGPPPRPSPPGVVASGVEAEPPPSLPGVEASGAGGGGCAPPPPPEKEASAA
PSPSPPRQVDDGGGEQEESATDDVGVDYAGPKTHPPSHN
>LOC_Os08g05950 22
MRCSCMVCLLLFFLLHLSVCHATSRNHRRLFAAAPAASPSTAALYGDDDITAAMHVWRSP
RRYMRAAAEEQRGSPAPAPLPDGALGLQPGGVEGAAAEARTTEEEAAGAGAAMAPFPAA
ADVGGKDDDDSGGGDGAADDAGVDYAPPKTHPPSHN
>Bradi3g16657 89
MQGRPAAMILAMAYLLLFFFFLHFSSSTHGANLPATAGDPLLSTMAHRRLLRSMMLQQHS
YVPDETTTMIQQLLGGPSPRPQPGGGVPVSPLGAAAAEQTPKMAPAPLPRGPTTADLITDD
RPGRGEVDIGVDYTPPKSHPPQHN

Group 5
>LOC_Os04g40640 26
MRLLPASGIVRLVALVFLLIFSSSLQQQAGVGAIRLHDRKQHGEQWEEERTQMRSFMTMD
YSSVRRRRPIHN
>Sobic.006G124200 73
MVSLGIVRLVALVLLVVFSSSLHQQAGVGAIRLHDRRQHAQQWVEERNQLRSYMTMDYA
PWHRRVPKHN
>Seita.7G141500 61
MALLGVARLVALVLLAVFCSSLHQQAGVGAIRLHDRRQHAQQWTEERDRFRSYMTMDY
HPWRRRVPKHN
>GRMZM2G022255 48
MVVGIVIRLVVAFVLLVLVFSSSLHRQAGVGAIRLHDRRQHAQQWAEERNQLRSYMTMD

YAPWHRRVPKHN
>GRMZM2G041513 50
MVSLGIARLVAFVLLVVLVFGSSLHQQEGAGATRLHERRQQHAQQWAEERNQLRSYMTM
DYAPWRRRVPKHN
>Bradi5g13666 91
MRLLLGIVRLVVLVFMVVVSSSVDQAVGVGAIRLHDRRTHGEQWAEERKQMRSYMTMD
YTPVHRRTPKHN
>Bradi3g47791 93
MRFLGLVSLVALIFLLSFRSLLHQQAFVEAFFPSVQGEGAAAIHDDSKKRQHAEEWAEDR
KRMRWFMTKDYGMPRRHTPRHNRLL
>GRMZM2G121160 41
MSPCPCIICIICSMHACAYSLLRQSEMRLVGLVSLVALIFLLSFRSLLHPQVLAGDGSAVTVT
SRRRDRSQQHAEQWAEERKRMRWFMTRDYASARRHTPRNNRLDP
>Seita.1G219800 56
MRLVGLVSLVALIFLLSFRSLLHQQVLVGEGAAAAAANGCHGRNRQRHQHAEEWAEERK
RMRWFMTRDYARARRHTPRNNRLDP
>Sobic.004G203301 77
MRFVGVVSLVALIFLLSFRSLLHQQVVVGEGSAVAAVARSGGGRHRHGSRNQLQQHAEQ
WAEERKRMRWFMTRDYASARRHTPRNNRLDP
>LOC_Os02g38330 30
MRFLGLLSLVALIFLLSFRSLIHQQVLVGEGAAASGFLHGSGDDGRRQHAREWEEERKKM
RWFMVRDYAHARRHEPRNNRLDP
>AT2G03830 100
MKLIRVTLFLCALAILLLVTPTSSLQLKHPYSSPSQGLSKKIVTKMATRKLMIISSEYVMTST
SHEGSSEQLRVTSSGKSKDEEKKLSEEEEEKKALAKYLSMDYRTFRRRRPVHNKALPLDP
>GSVIVG01003987001 196
MICLSLLSQSQTLAARPHFSGLMGLQDPPKQIRRLVKDQLPVPWWWSEDYTQARRRQPV
NNKLDP

Group 6
>Glyma.02G122800 151
MELIIIIITLLSFSFSYLIPTCSSSQIQLEPSLQLEDDNIGVNTNLQLSLPTVPRKLRFTEKVKED
DGARDLASHKQENSFAAAGKQYHRKQNMVLGKKGTRQEWMEVDDPSQYFTMDYTRV
RRRRPIHNKQLPVNP
>Glyma.01G065000 161
MELIIIIITLLSFSFSYLIPTSSSQIQLEPSLQLEDGNTGAVVNTNLQLSDLPTIPRKLRFTEKVK
EVDEARDLASNKQQEGSFASGKQYHRKQNMVLGKKGTRQEWMEVDDPSQYFTVDYTR
VRRRRPIHNKQLPVGP
>Medtr5g048090 137
MELIKIITFLCLFFSSLLVESHPSFHHQDQQLSLPILHRKIMFTEKVKESDEARHLVSHKRKD
SLPAGKQNMEVGSKGTRQEWMESDDPSQYFTMDYNRVKRRRPIHNKQLPVGP
>Cpa.g.sc1.170 199
MEKIVIICLACMSLLLLLIEPSSSLQLIHLQIPLYQQDKVFLSRKLKFTDEVTVKQHESQDAT
LNTDQHKRLPSGKAKQKEESMKESGRQGRWIEGADASQFFTMDYTHVRRRRPIHNKSLP

VAP

Group 7
>Sobic.006G112000 74
MVSFIRRTSAMGLVVVILLIVSLLTIHLPAACARHVVVLNPDNNGLNNRGNKNLGKALAV
TDDDDLAANKVISGRKLGAPNNKEGTKATVGATTTAAAAAATSAGSRPRTVKMRAAAR
HGDAVTEMYDMLRRDYASRASRRRPINNGATPLQVNKKP
>GRMZM2G050458 42
MMSCTRRTSAMNLVVVTLLIVSLLTVHLPAVACARQVAVLNPYKNELDNRGNKNLGAAL
AATASTDDDDGLAASGKVFPGRKLGAPNKEGTKATVGATTTTSAAGSRPRTVKMRAAAR
HGGDEATEMYDMLRRDYTSKANRRRPINNGATPLQVNKKP
>Seita.7G129500 65
MSRRRTSAKSLLALVLLIIVSLLTLHAPIAFARHVVVLNPNDGVNNRGDSKNLSKVLASAS
TDDSAANKGVFSGRKLGAANKEEGITKATTGATATATASAGSRPRTVKMRAAWKHGDAA
AEMYDMLRRDYAWKASRRRPINNGATRFQVKKP
>GRMZM2G364839 38
MSCIRRTSAIMSSLAVSILLIVSLLTLHLPIACGRRHVVVLNPSDNGMMNSRGKKVSSTVG
AAAAAATTRTVKMRAAVKHGYAVAEVYEMLRRDYASKARRRRPINNGATPLQVNKKP
>LOC_Os04g39230 23
MSSRRTSAANLLAVALLIISLLLLPLLHLPVAHARHVAVLKATDSSSAISIRSGHVEPTPASGA
VQRRPASSGASNRGGGGRRRRAAASSRSTVEMRASAWAKHHRDEVARMHEMLKRDYAS
KARRRSPINNGEPSLEEEDLP
>Bradi5g12825 83
MSSRSSRSTSTVVGLLVVALLFSTFLAPHLPAASARHVAVFKAKDDATGQAEAAGGRSSRP
GRTSTVEMRGGDAWAAAEMQDMLKRDYAYKARRRSPIHNDEPLQENDEP

Group 8
>MDP0000215329 189
MEFSYCLVFVLVSAASIWNISANSRHQLGENAVVPNDVSILKGEDIDHHQVVSPPTATKED
KRIGGRKMAAARLEKDVKVVAEGGVSGTRRSNSSDLEGQSSCGNVECRQSTTESRFSEKI
VDHDHEHAGFVAFSQDYNSPRHHPPKNN
>MDP0000322433 181
MEFSYCLVFVLVSAASIWNISANSRHQLGENAVVPNDVSILKGEDIDHHQVVSPPTATKED
KRIGGRKMAAARLEKDVKVVAEGGXSGTRRSNSSDLEGQSSCGNVECRQSTTXSRFSEKI
VDHDHEHAGFVAFSQDYNSPRHHPPKNN
>MDP0000259300 185
MKFSYRLVFVLVSAASIXNISANSRHQLGIYFPFFFCVIVHVFPKXLKKGENAVVTKVVST
XKVQRQDIDHHEVVSPPTATKKDXRIGGRKMAAARLEKDVKVAVDGPIRGTRSSKTLSSS
SDLEGKSSCGNVECRESTTASRFSEKSVDHDQEYAGFVAFSQDYHSPRHHPPKNN
>Medtr8g066030 144
MACILLVLLCASWLSNARLHPQGGVVEVNGISSTKANVTTAIDGKNESVNSPSRSSFHGK
RSLRTHLSDDSGFVAFTTDYHPPRHHPPKNNK
>Potri.008G112400 130
MAASPVKFTCLLLLLLFTSAWLNSARAHPTVEVDTFSSERIIVDGDGMVTTSIKKIGGRKV

VRAHNIKRPDIKGNVAHGGTSRISSANDQVVGKYGYEREVMDIGNKRNGGRFPEGVKES
GFVAFNADYHEPRHHPPKNN
>Cpa.g.sc998.2 203
MKFVCFLIILLFSTLPTPSFAVELHPADENTGTGVENVKIVGNRKMYVDGVMKIERKEITES
VIDKRGIMRMMHEKCKKAGNHCEEKSDDGGFVAFNADYHAPRHHPPKNN
>Glyma.02G124200 172
MVLIKKSACVVLVFLCAACLSTARLNPKGASVNDIPDIKASIATSVTKSTMAAIDEDNLNR
PSEKRLMSKSGKVISAPNVEAKRNNARSCLSDEVNEDPLAFTADYRRPVHHPPKNNK
>AT1G13620 96
MTNITSSFLCLLILLLFCLSFGYSLHGDKDEVLSVDVGSNAKVMKHLDGDDAMKKAQVR
GRSGQEFSKETTKMMMKKTTKKETNVEEEDDLVAYTADYWKPRHHPPKNN
>AT2G04025 102
MTTLSKILCVLIILLLCFSFRYSLHEDGNQQSSRDFVSTAKAIKYGDVMKKMIRGRKLMM
ASGEKEEAETKMKRGNRETERNSSKSVEEDGLVAYTADYWRAKHHPPKNN
>Solyc04g005240.1 112
MLVPRRILSLLLILILIAQILPNTTSNYQGGKIATKRSNSVSKREALANTGGEQILSRRPLSQS
GGKDINAEYVAFTADYKSPRHHPPRHN
>GRMZM2G096431 40
MAPMFCPRAGGVEANRNAETMARMLLAGKSQKTVLAVSPELLSVTGSKGGADIKLELSV
GKIRTGEETARSNVLTGRRLAFGDAVMEQKETENSGIRTDSGAKYAVGKCGHGGGKDLS
MNCSKHTPKVYFDGHIPFTADYRSPRKHPPKNN
>Glyma.17G082400 159
MVSARSTTFLLVFILLPLRFSSAHTQHQGKKTPVVAKLETKRSPVKVEIHHGVPAVAETDQ
RNRLLPGRKMMSKGVISKLINAVDSDNGINMAKGVTTKGKARCLNGNCKRETGKRILGL
KHKDRKRSNIGRSTKAKMSGLVPLNADYYVPRPHPPKNN
>Glyma.05G017600 173
MVSARSTTTFLLVFILLPLRFSSAHTQHQEGKKTLVVAKLETPRCPVNVEIYHGVPAVAETN
QGIRFLPGRKMMSRGVLSKLNVVESENGTNMAKGLTTKWKALCLIGNCNREKGKRILGL
KHKDSKRSNGRSTRVKMSGLVPLNADYYVPRPHPPKNN
>MDP0000620898 178
MASFKLITFVPVFILLHLIQSTAHTHGAMNKVVLAADETGHLVVLRRSLVDVEXEIHDGVP
AAVEEAEDTRIGGRKMMPERNLGMVKVKVLKSNGERRLRSASSSGRVKVKIPGFIPFDAD
YHVPKPHPPKNN
>MDP0000295005 190
MASFKLITFVPVFILLHLIQSTAHTHGAMNKVVLAADETGHLVVLRRSLVDVEXEIHDGVP
AAVEEAEDTRIGGRKMMPERNLGMVKVKVLKSNGERRLRSASSSGRVKVKIPGFIPFDAD
YHVPKPHPPKNN
>Bradi4g32950 88
MRCAGSVLLLLLLLSLSAALTAEANMEGLNGYNAAPSLPGRRLLRERRAMAALAHGGGGP
EKKNEVVEERKGEENTVANKAHVHGGEKTVEVTVVGLSGESSGKKNEGSKRKFAGQVP
LSSDYRIPKCHPPRNN
>Spipo12G0015500 82
MAALSLTGFLLRLLLFSIALRQAIADDRRVTETGAIADIDRALERSPVSLLSTFTTGIFLLFV
SRLIGFSRYQQRRGHVVARAGPEANGMKRMEGGRRPGVDCGRREVGKLNVECALRGRR

RHSGVNFVDFIPFSSDYRGAKTHPPKNN
>Zosma93g00670 80
MVIATNLTRLMILYYLFLSSLSETTGAIEKAPMHPQDCTNKENMQNLWGSHGSRLMGGKR
SDAESLSDVIVVEVKEETKKKLKVKKHSTTANCRYHRKGMLNVECELRDVVPSIHYNLPP
FTSDYRDPRTHPPKNN
>Sobic.010G199500 68
MTDIPKSRAKQLVMQPLNCISFLLGLAILSATLGPFVAIAHRELLSVTGSKGGAEIKLELSV
DKIRTDEEVRSNVLTGRRMAFGNAVMEQKETKNSGSRTSSGENKNYSTNSHVPSNIKDSS
SSGMQAGPSRNRVKLEGSTSVTALSNPNPQHLRTLPSKHLRNSNSGSKNELRDSVVHRTL
YKEKMLEASDEVLKFLNKDYHASPHKRKPVHN
>LOC_Os06g42754 28
MTDIPKSRVRQLVMQPLNCVIFLLGLAILSVTFGPFVTIAHRELMMATYSEKGPENKLDHG
VDETRTYEEIKSKIFAGRKMAFGVAVMENHPKDARSKPSSGEISNYSSNSRVPSSLKDSSSS
TMKARPSVDNIKLEGSTSEQTLNIPNPQHIIILPFKPYYRHLSLGSKKEQKGSSICCNSYRTN
EDWKEKMLESRDEVLRLLNKDYHANPHRRPPVHN
>Bradi1g35510 84
MSTRHYKSTVLCCAFSFPFLSVTRQTYQRTVPPLVMRSLKCVSFLLWMVILLATIGSFGTT
AHRELLMATRGVETKLVRTELTVEKTNIDEEIRNNVPTVRKMDFGTADANDARSIPSSGDI
KNNSTNSCAPSSVIKDSGSSRMKARPSMGSIKLEGSISGQVLNVPNPRHIRILPSRNPSSGSK
TELEDSIVPSTLYKNNDDRKHKMLEASDEVLKFLNRDYNRFPRRRTPVHN

Group 9
>GRMZM2G065781 53
MRRQLKCVGLLLLLSLLLPALSPVVVVATARRELLPLMATGDEGRPQDVSSSGTWMPPGA
AEEAVVRRKDEAMKTNGRRFRRTSSWNWKKMPAAASQVSFGGRIPFTADYHSVHRHPPT
HN
>Sobic.004G074100 72
MRRQLKCVVLLSLLLAALSPVVVVVATARRELPLMAATGDEGRQDVSSSSGTATAEGGAE
VVIVRRRDEEAVKNSRRRFRRTSSSSSSGKQMPAASPQFNFGGGRIPFTADYHSVHRHPPT
HN
>Seita.1G055100 64
MRRLISCVGLLLLLGALLAALSPAVVVATARREMLMAASRDEGRGRAAAVNILGNAPPPPP
ASANGGEDVIGRRKDEAVVARSHRRFRTRRIPASGSQVQFGGRIPFTADYHSVHRHPPTHN
>LOC_Os02g09760 33
MQQLNWVSLLLLLFFMVAALSSFVATAHRELPMARKVDEIGDHLQAKLDNQASSVSVTR
ATAKAEHDHQEAVMRKCKNGRKNCKNFRTRKLPADADGKIHFDGHMPFTADYHSVRRH
PPSHN

Group 10
>Seita.2G241700 66
MRCAAVLLALLLSLSALSASTAEAHEERLDDDAVLLSGRRWLRGRRIIAATGHGDAGKKD
EVVEGKGAKSTGADQEADAPAEAVHDSGKRSKGSATHAMFQAPRQGDTAAVIPEVLGM
DYNYKLDARHHRPINNDAPLEDLAKKP
>Sobic.002G235800 71

MRCAGVFLALLLSLSALSASTAETHKERLGDAAALLTTGRKWLSGRRTLADARKDEVME
QGRRGAKSTAGTNQEADAPAEAVHDSGKRSKKGSATHATFQASSRHGGGGSDASEPEILG
MDYNLNLNPRRHRPINNDAPLGLGELAAKTP
>GRMZM2G137551 36
MRCAGVLLPLLLLSLSAASTAEAHKERLGDAAAALSTGRRWLTGRKTLAAPGRDEVVEG
AKKSTGANQEADAPDLRKLYMARVICLYCQAAISFQCKRSEGPVTHATFQAPRHAGGGG
GGTDTSAPEILAGMDYNYRLDARRHRPINNGAPLVELAAGTP
>LOC_Os09g29870 21
MRCAGVLLLLLLLLSLSALSASASEANEERLLRENAMPITGRKWLRGRKAMAAAGRLGHG
GVVVVEGKGGGEEKNKKNTGANTAHVHGNGSERAVDVAVVGKSGGKHGIKSPLNEGLK
CVISVVPGAANQEADASAKAVNHEEPSKHDDTAAAVSRMMSMDYKTQDARHHRPINND
APLDHELVEKP

Group 11
>Glyma.08G253200 156
MKRRNTDQMMKITLLSTLLLGCLLVMTVQGDSSAGVVPAVTVVEEGNKNKLGVSSQKEP
FVRSGAGHVRKLGLGGKKFGTNEVTSVDSKNGKGAYGGETSKISGKDDDGLKKSSGSRD
QKNDDQKLMMMGPKEYLKSTKFFVVPRRIPTSTYPKSCSQDCIIAVPVKSSLGSSSSSHEH
QISEETPQKDETQRFADAAKEIANMMNKDYHGRPSHRPPINNNEPRN
>Glyma.18G275800 168
MKRRNMDQMKITALSIFLLGCLLVMAVQGDSSARVLPAVTAVEVGNINKLGVSSQKEPFM
RTGAGHHMRKQGLGGKKFSTNEVTSVDSKNGKGADGGETSKTSGKDNDGLKKSSGSRD
QKNDDQKLVIMGPKEYLKSTKFVVPRRIPTSTYPKSSSQYCNIAAPVVKSSLGSSSSHEQKI
SEETPQNSTQKDETQRFADAAKEIAYMMNKDYHGRPSHRPPINNNEPRN
>Medtr7g017380 146
MKFIFILLSSLLLGGLLILTFQRDSLARVVSGQEWNKNRLEVAEITAISSQKEPFQRITGFFH
MRKLGFGTIMHHEETSVDSKNGKRETSKISGNENDGLRKSFRSLFQVKMNDAQKHVTM
KANTKFAIPRNSLTNTNTKCSQNCDVVPIKGSPERSTRSIQISEEAQSIDAAKEIGSLMYKD
YSHKGKPSHRPPINNHQPNNP
>Potri.004G112600 131
MVYKRFLGLALGLLFVMNAIWGSCAYDYHDGEMKKKEIVGFGENTVLSVDKELILSGFS
TASTRKMGLGGRKMAVQKESRRETEKEQGLHGQASEDNSGANYSNVKCDFQVKGSDFN
QKDMSNLERETLSARLGTPRTDQTVHLPKSNSKDSKALPTKTSLESPSRADIDQEPQGSAT
DLKSEMQRLLDATREMVNLMNKDYTIKPPKRKPPINNKEPIH
>Potri.017G102200 133
MVYKRFLVLVLELLYVMNAIWGSYAHDHQDWEMNKYELLGPEEIDVPAHKELNGFSSTA
STRKMALGGRKMAVQKVTRREIEKEQGLHGGAPEDNSGANYSNVKCDFQVNGDFNVRC
KLRAKRNSPKMIKGPGNEKNALDESLGGSNDQLNNIKQKDMNNLERKTLSARLGTPRTE
TVNLPKSDPELSQDSKALPTKTSLESRPPRTDMDQEPQGSSTLPKGEMQRLLDATKEIVNL
MHKDYSGHARPGRKPPVNNKVPIH
>Cpa.g.sc14.249 197
MADCGKLSDGTRSPRKVLQDRVNDKSGVLETEPLKSVGLRTPRRSSMSDQQEETDCLQT
SAEAKRLLERSKEIVSLMHKDYSGMSKPRRKPPINNNEPWH
>MDP0000264944 191

MMLXLTSLVVGLLFGMXALGQFSTAHAHQGKEDISMTGKRAVLPDQKEPNVDGISIAGSR
MIGLGGRKMVAHRVLRKSEPVRPDGQQNEEASTTSKNPGANRSTSDGNCNFEGREDLNV
KCKLGDRDKSKDNPKASSNFSGKLQNQMNQQDXVKILELTTLKSTSAGSLEIPRDLIPNHS
EDFKTKHPTTEAQMTKSYDEKDDHKLTDNEARSLLKATREIANLMRKDYHHQMGRRKP
PVHNLQPRP
>MDP0000280679 180
MDGSGESSESVSAXTNSKSMGNGVSSVDDRCPKQVSPLRGGGSRNTSPVGCVRSRNTSPS
RQKVVKTKPRGLDEETASTFGKPVHPDVQMEDNIWAMLPEDLLNEILARIPPFMIFRLRCV
CXRWNTILQDRSFLKFHSQVPSHGPCLLTFWKNSQTPQCSVFSLPLKTWYRIPFTFLPQWA
FWLVGSSAGLVCFSGLDGLNFRALVCNPLTQTWRTLPSMHYNQQRQLIMVVDRKDRSFK
VIATSDIYGDKSLPTEVYDSKLNTWSIHQIMPAVNLCSSKMAYCDSRLYLETLSPLGLMMY
RLDTGYWEHIPAKFPRSLLDGYLVAGTXKRLFLVGRIGLYSTLQSMRIWELDHAKFVWVEI
SRMPPKYFRSLLRLSAERFECSGQDNLICFTSWNQGKGLLYDVDKKVWSWIAGCALQSY
NSQVKAKKKLNTMMLQLTSLVVGLLFXMHALGQFSTAHAHQGKGDISVTGKHAVSPDQ
QKCKKLSGEYXNSERFSCSIXFKXIYKLTHISMQEPNVDGISIAGSRXIGLGGRKMVAHKV
LRKLEPARPAGQLNEEASTTSKNSGANRSSSDGNCAFEGKGDLNVKCKLGDRDKSNTSTT
SSKKYSKARIILKLQAIPMEKPQNRLNQQDDMKILELTTLKSTSAGSLEIPRDLKPNHSQDF
KTKQPTTKAPKTICYDEKDDHKLTDEARRLLKATREILCVLPKFLSVEKIFIVPRTRSSKNE
LSVTGNGPSDQQEIIEHGISTAGSRMIGLGGRKMVAHKVFRKLEPXRAVGPNRDTRSSTTS
TNSGKESADACGETTKYDETDDPKLTEETRRLLKATREALNLMTKDYQGRDRRRKPPINN
HEPQH
>MDP0000287307 186
MAGTCCELIWLRFHRFVSEGSYVYSPFAGKNELSVTGNGPSDQQEIIEHGISTAGSRMIGL
GGRKMVAHKVFRKLEPXRAVGPNRDTRSSTTSTNSGKESADACGETTKYDETDDPKLTEE
TRRLLKATREALNLMTKDYQGRDRRRKPPINNHEPQH
>Solyc03g114700.1 113
MVSLSFSSFMLVMLLFIFLTIKDTIAHTDDQAKQIVHEAENYNASDLKSYLSVKKKVLESV
PAAAAFRLGGRKMMIERTKDMKEVKKGESSGAASASVHSVGNLNYRKGQGKLREMSGN
YNRPLGHDSRKNLHYQGNESDSMNSASTHNLKNDDQSDDNGSFQKIESEKLVDDMTELF
TMMNKDYVGGPGSGSKPRHKPPINNFQPLHRSNP
>Solyc02g089300.2 114
MVSLGFNIQLVLVIALLILQPQQHTSANVLFGKLRKKEWLKFTSIEKDHDSHVDGIVASAG
GIRKMLVYQFSVKGDAGFDSKKTSHMNVQADIKKNHEIGKKEESKEFVDVEDEITKLLTK
DYSGRSKPRRSKPPINNHKPTD

Group 12
>AT3G02240 103
MEMKKWSYANLITLALLFLFFIILLLAFQGGSRDDDHQHVHVAIRTKDISMGRKLKSLKPI
NPTKKNGFEYPDQGSHDVQEREVYVELRDYGQRKYKPPVHN
>AT3G02242 98
MKKWSYAKLMTSALLLVFLSIILLAFHGGSRGDNHLYDHVAIGTKDILMGRKLKDLKPKT
ESLKMINPKKKNGFEYSDQVSSDLSRQEVFVDMMARDYQGPKPRSKPLKNN
>AT5G15725 97
MKKTSLKLMTLVLGFCFVIYLLQGPRGGSRNGDLLIARKLISLEPIETKNAARSLKDSISTD

LEEEVDRLMEHEYPSPVKPRKRTPVHNGVRNRH

Group 13
>Glyma.13G102700 177
MMSSSIFLLCCLLIFSLYSLSQHTGYSLDATPTRKLSMPAPVHDHKTQHKMQGAEKAMES
TPANEDSMTPDYTSNSYSPHDLVFHIDYHGVTTHPTPKHPKP
>Glyma.17G056400 174
MSSSIFLLCCLLIFSLYSLSQHTGYSLDATPTRKLSMPAPVHDLKTQYKMQGADKAMESTP
ANEDSMTPEYNSNSYSPHDLVFHIDYHGVTTHPTPKHPKP
>Medtr4g126770 148
MGSKPDFHHMMSSSIFLLCCFLTLSLYSISQHTSNTYSLGFTPARKLSMSAPGHDHKFHET
QVSDEAMESTPANADLTTLEITGNSYSPHDLVYHTDYHGVTTHPTPKHPKP
