# Supplementary material for: Evolution of RGF/GLV/CLEL Peptide Hormones and Their Roles in Land Plant Growth and Regulation
Source: Int J Mol Sci. 2021 Dec 13;22(24):13372. doi: 10.3390/ijms222413372 (PMC8708909; doi:10.3390/ijms222413372)
Supplement: Supplementary file 1 [file ijms-22-13372-s001.zip › supp/Supplementary.pdf]

International Journal of
Molecular Sciences

MDPI

Supplementary

Evolution of RGF/GLV/CLEL Peptide Hormones and Their Roles in Land Plant Growth and Regulation

Yitian Fang [1,2], Jinke Chang [2], Tao Shi [3], Wenchun Luo [2], Yang Ou [2], Dongshi Wan [2,*] and Jia Li [2,4,*]

1. State Key Laboratory of Microbial Metabolism, School of Life Sciences and Biotechnology, Shanghai Jiao Tong University, Shanghai 200240, China; fangyt@sjtu.edu.cn (Y.F.)
2. Ministry of Education Key Laboratory of Cell Activities and Stress Adaptations, School of Life Sciences, Lanzhou University, Lanzhou 730000, China; kingco_chang@163.com (J.C.); luowch12@lzu.edu.cn (W.L.); ouy@lzu.edu.cn (Y.O.)
3. CAS Key Laboratory of Aquatic Botany and Watershed Ecology, Wuhan Botanical Garden, Chinese Academy of Sciences, Wuhan, 430074, China; shitao323@wbgcas.cn (T.S.)
4. School of Life Sciences, Guangzhou University, Guangzhou 510006, China

* Correspondence: wandsh@lzu.edu.cn; lijia@lzu.edu.cn

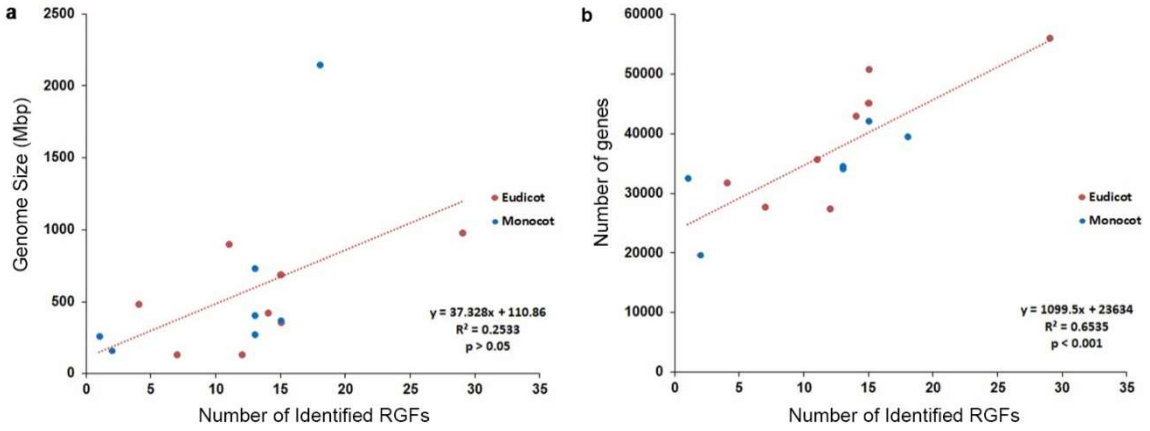

Figure S1. The relationship between genome size (**A**) or the total number of genes within the genome (**B**) and the number of identified RGFs from all the monocots (blue) and the eudicots (red) from the selected 24 species.

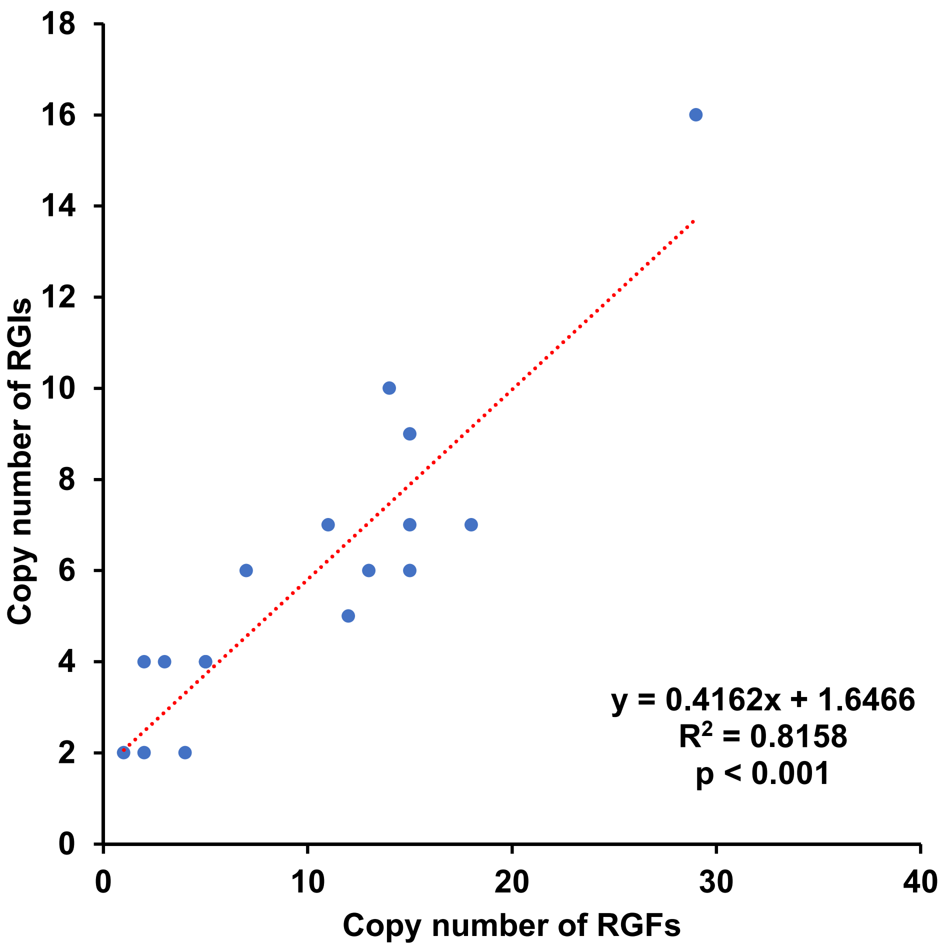

Figure S2. A significantly positive correlation (Pearson correlation, *P*-value < 0.001) of copy numbers between RGFs and their receptors, RGIs, across plant species indicates co-expansion between RGFs and their receptors, RGIs during plant evolution.

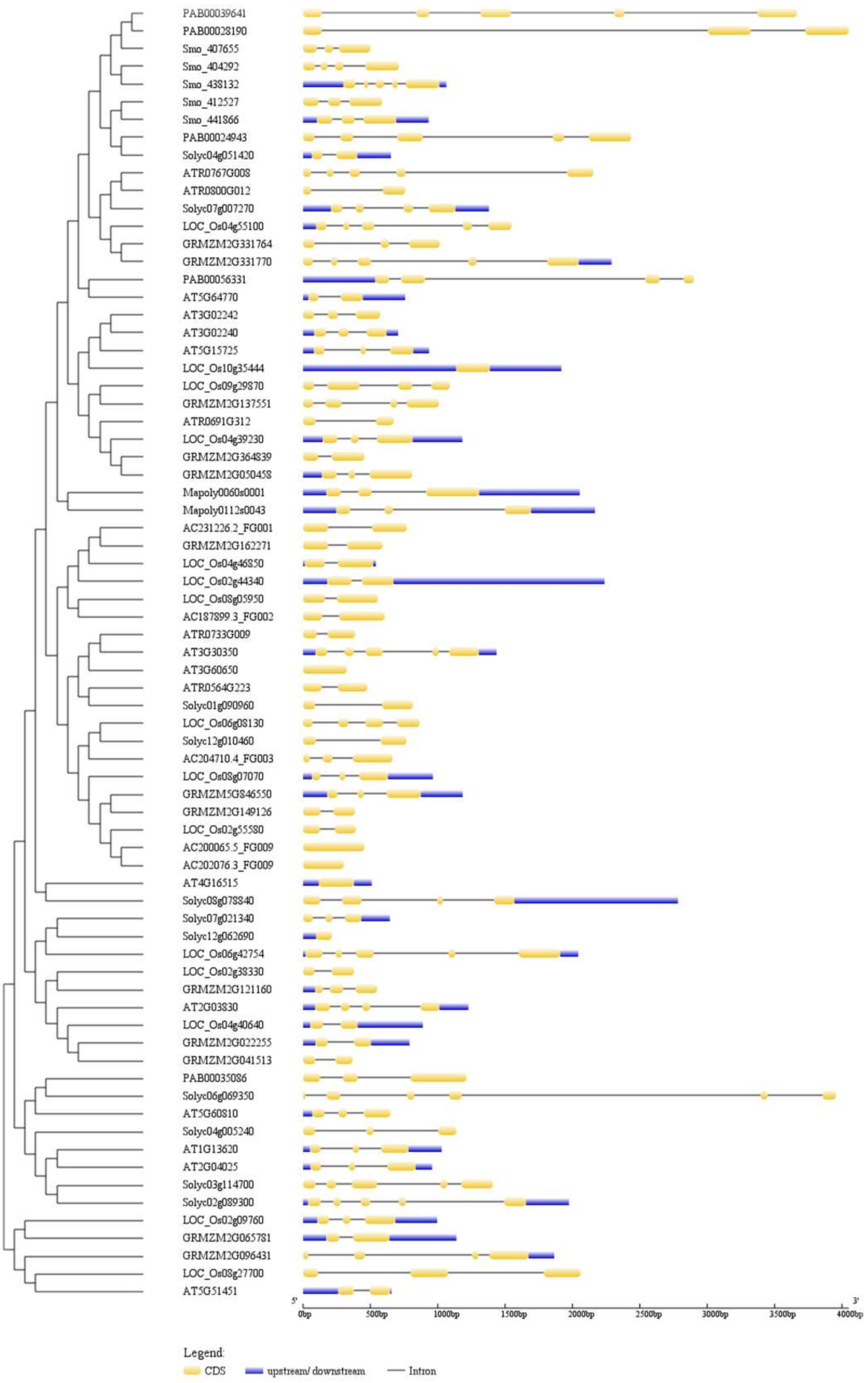

Figure S3. Diversified gene structures of the *RGF* family. The intron/exon structures of each gene were determined by comparison of the CDS sequences with their corresponding genomic sequences. Phylogenetic tree constructed by the neighbor-joining method with 1,000 bootstrap replicates using MEGA 6 software.

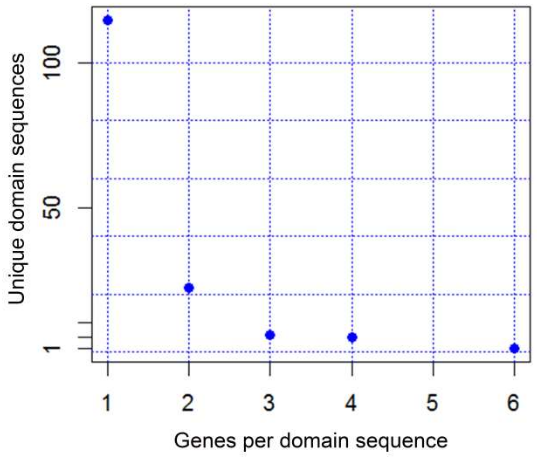

Figure S4. The distribution of the number of genes associated with each unique domain sequence. 115 RGF domain sequences were present in only one RGF protein while 34 were shared by multiple RGF proteins.

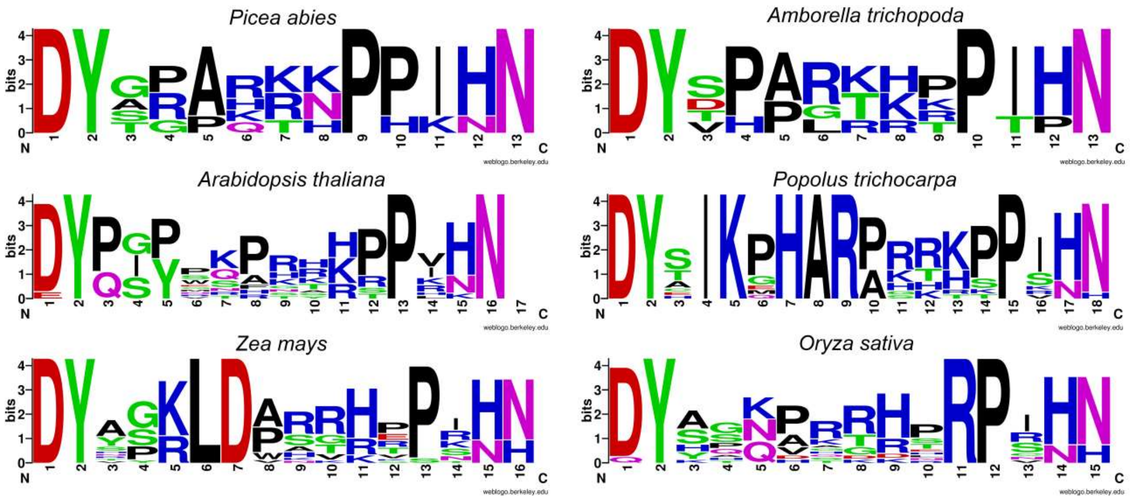

Figure S5. Sequence logos for the RGF domain in each species. Sequence logos are based on weighted RGF domain sequences identified in the genomes of different plant species. Except for the conserved Asp at position 1, Tyr at position 2, and Pro in the second half of the RGF domain, the other amino acid residues differ greatly. The size of the amino acid symbol represents the degree of conservation.

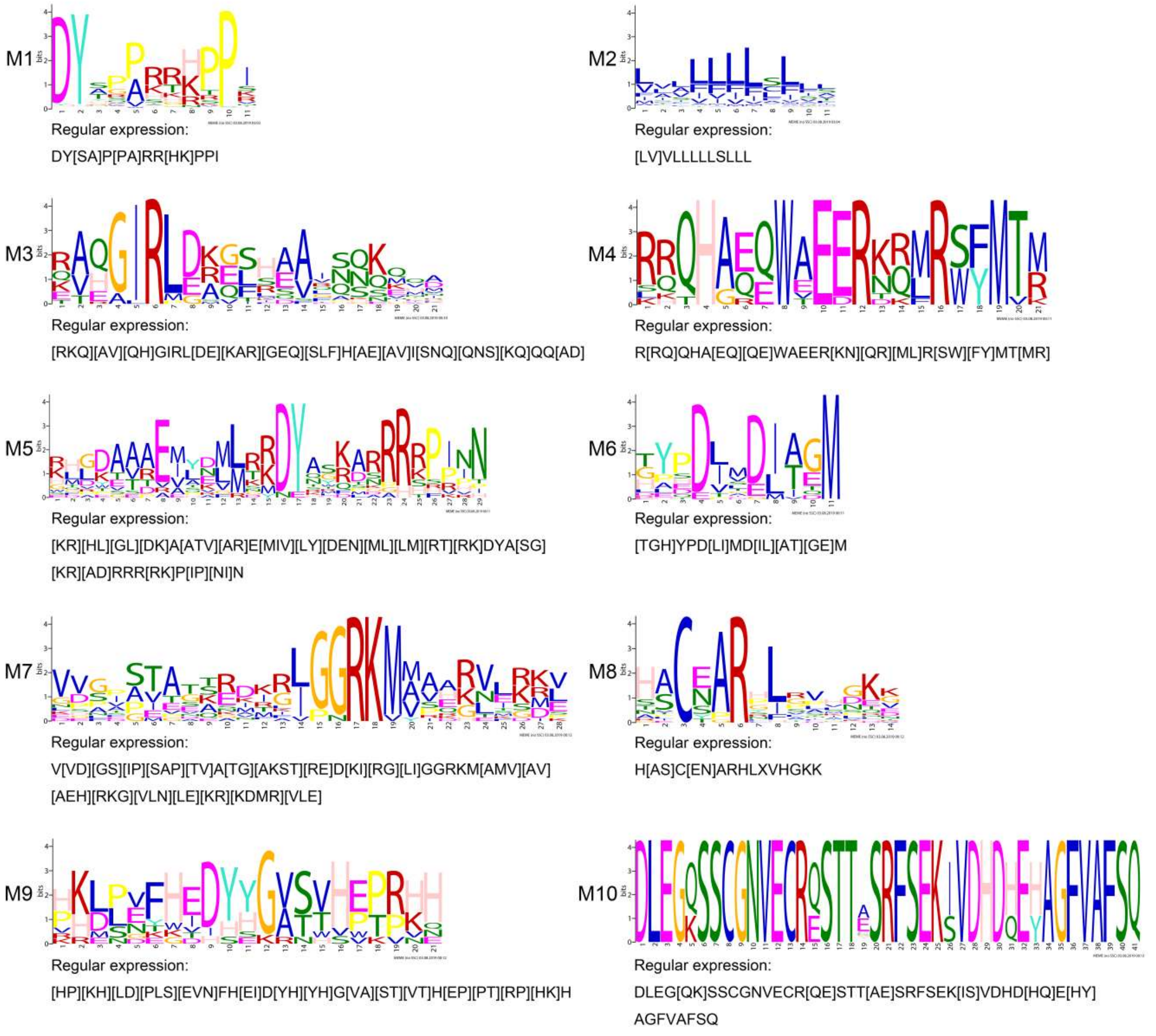

Figure S6. Sequence logos of different motifs identified in the RGF family. Ten distinct motifs were identified in these proteins using the MEME motif detection software. Most RGF members contain Motif 1 and Motif 2, and some motifs are unique to only some lineages. The size of the amino acid symbol represents the degree of conservation.

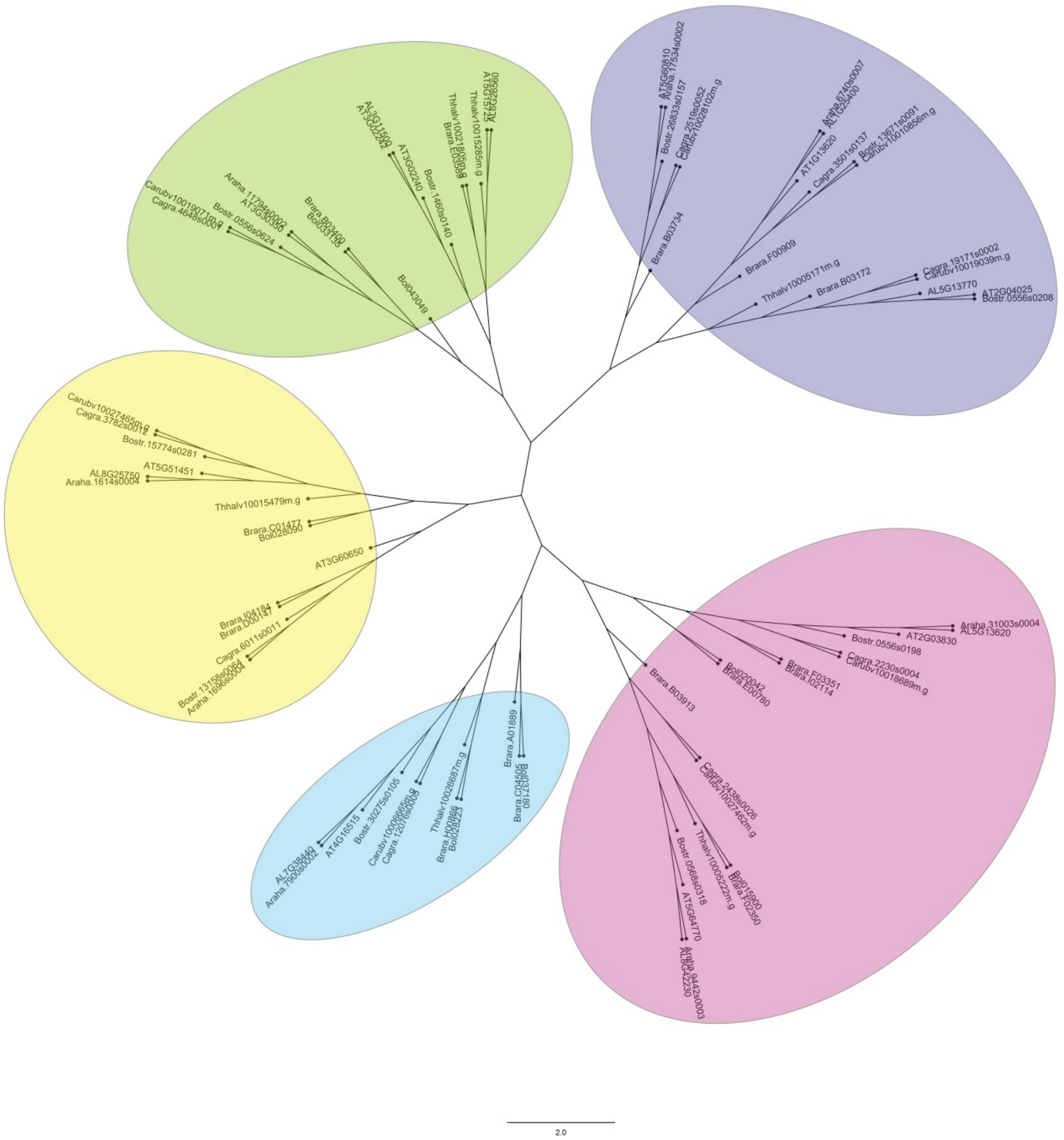

Figure S7. Phylogenetic tree of RGF proteins from Brassicaceae species. The phylogenetic tree was constructed using MEGA 6 by the neighbor-joining method and was based on full-length amino acid sequences with sequences from some Brassicaceae species.

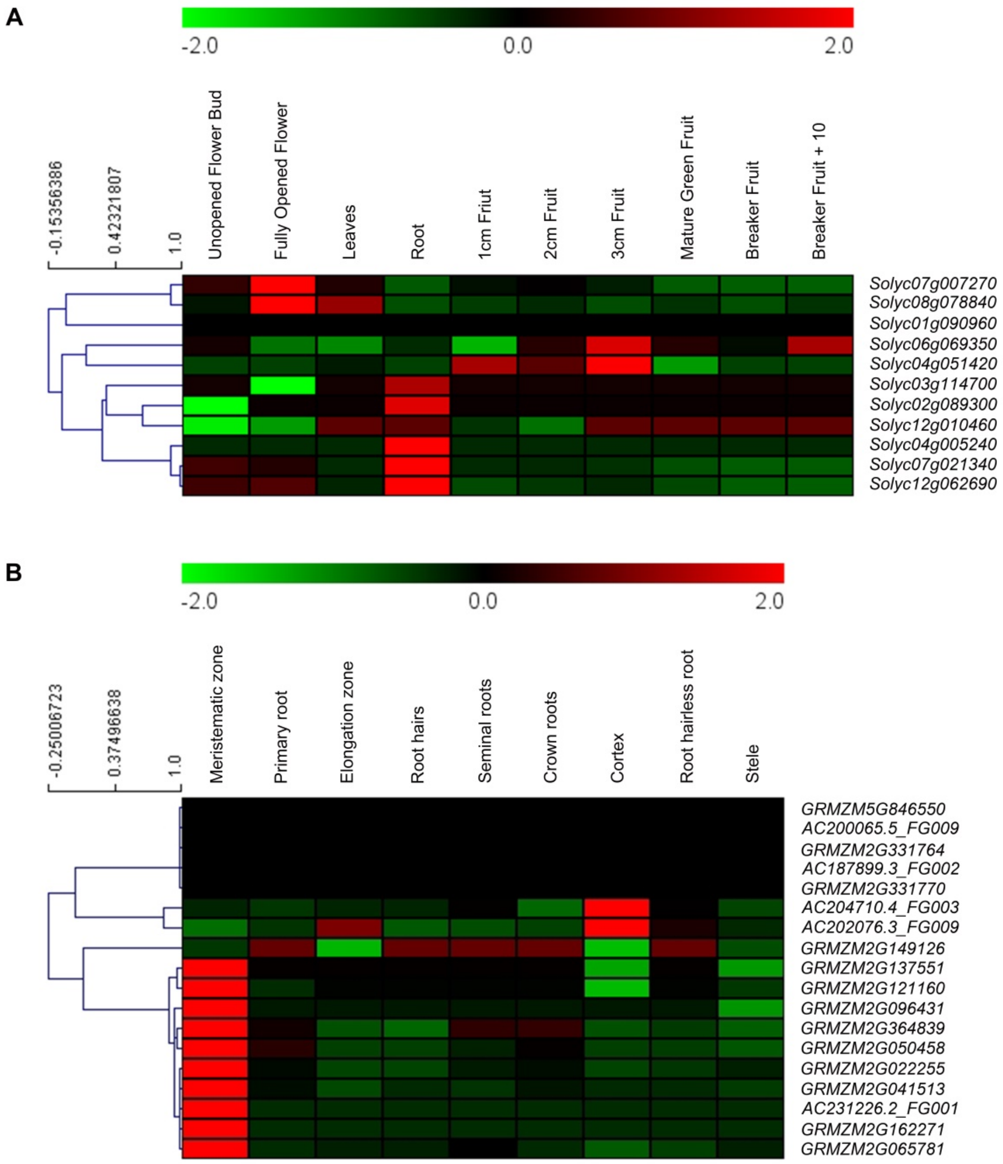

Figure S8. Expression analysis of tomato (**A**) and maize (**B**) *RGF* genes. Heat maps indicate the expression levels of *RGF* genes in different tissues as determined from the e-FP browser. The color scale indicates the expression levels of *RGFs*. Red marks high expression, and green marks low expression.

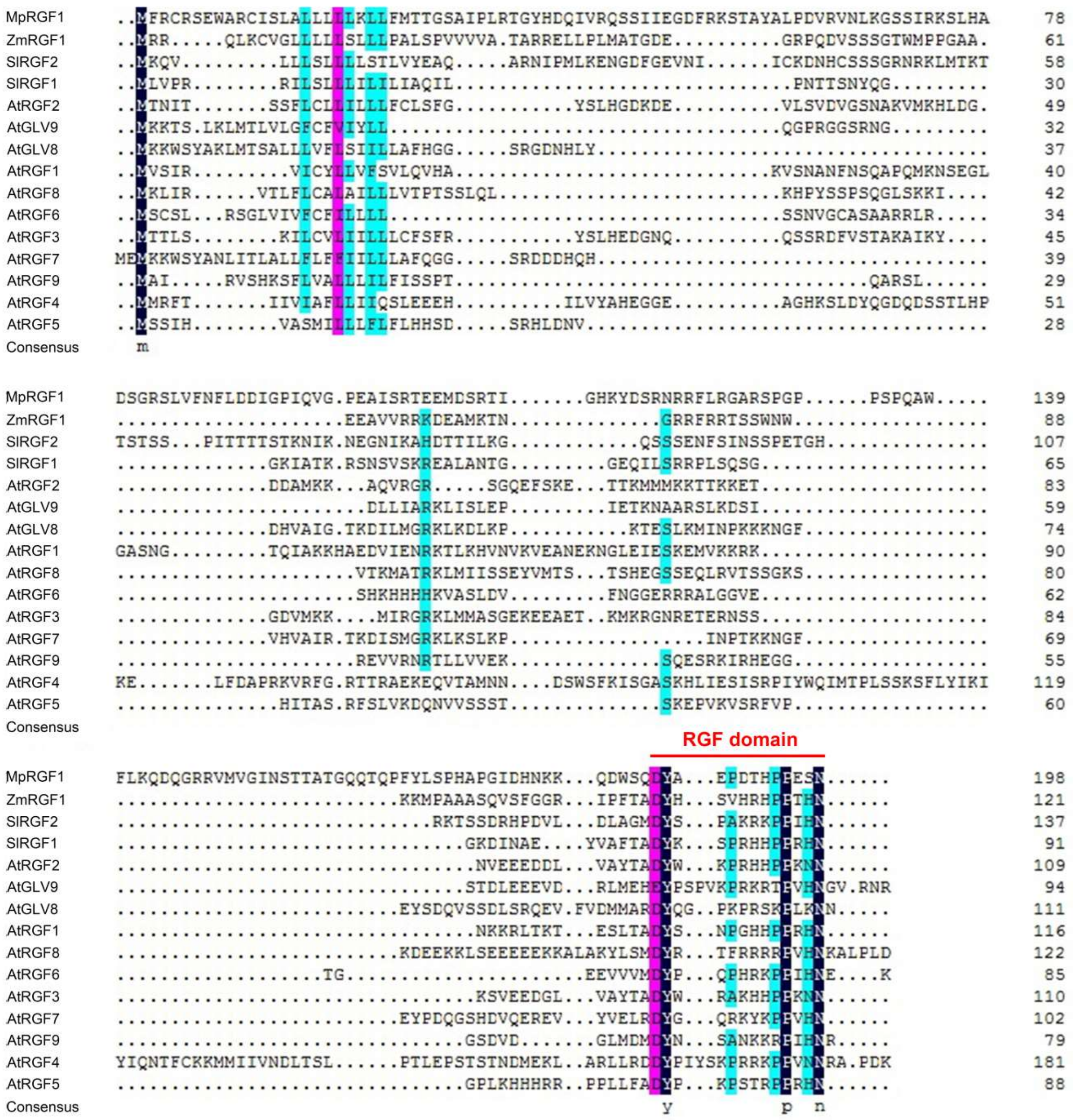

Figure S9. Multiple sequence alignment of some RGFs. Alignments of full-length sequences between AtRGFs, MpRGF1, SlRGF1, SlRGF2 and ZmRGF1 were generated using DNAMAN. The colored boxes indicate certain key basic amino acid residues.

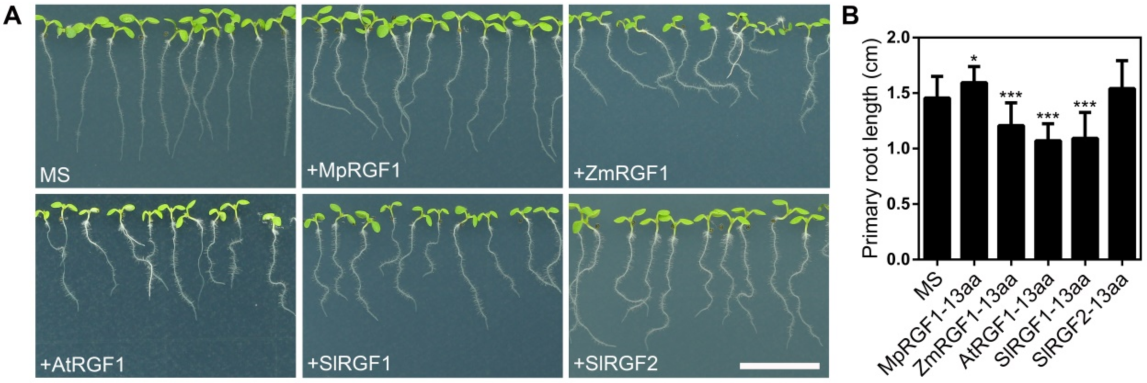

Figure S10. Root length of Col-0 growing on MS medium without and with the treatment of synthetic RGF peptides. (**A**) Six-day-old seedlings of wild-type plants grown on MS medium or on medium containing 1.0 μM of synthetic peptide. (**B**) Statistical results of (**A**). Student's *t*-tests were performed. One and three asterisks indicate the values are significantly different from the control at $P < 0.05$ and $P < 0.001$, respectively. n > 30. Scale bar equals to 1 cm.

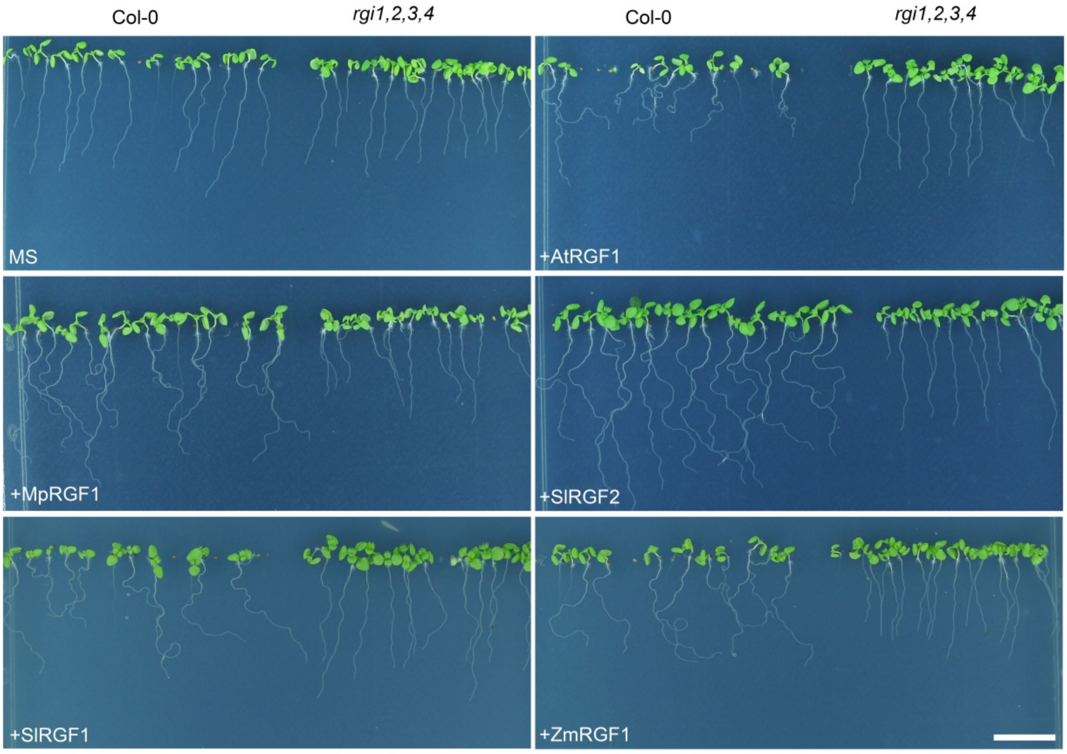

Figure S11. The synthetic 13-amino-acid (aa) peptides from RGF motif triggered a wavy-root phenotype when seedlings grown on slanted plates. Nine-day-old seedlings of wild-type plants and quadruple mutants grown on 1.5 % agar plates inclined at an angle of approximately 45°. Scale bar equals to 1 cm.

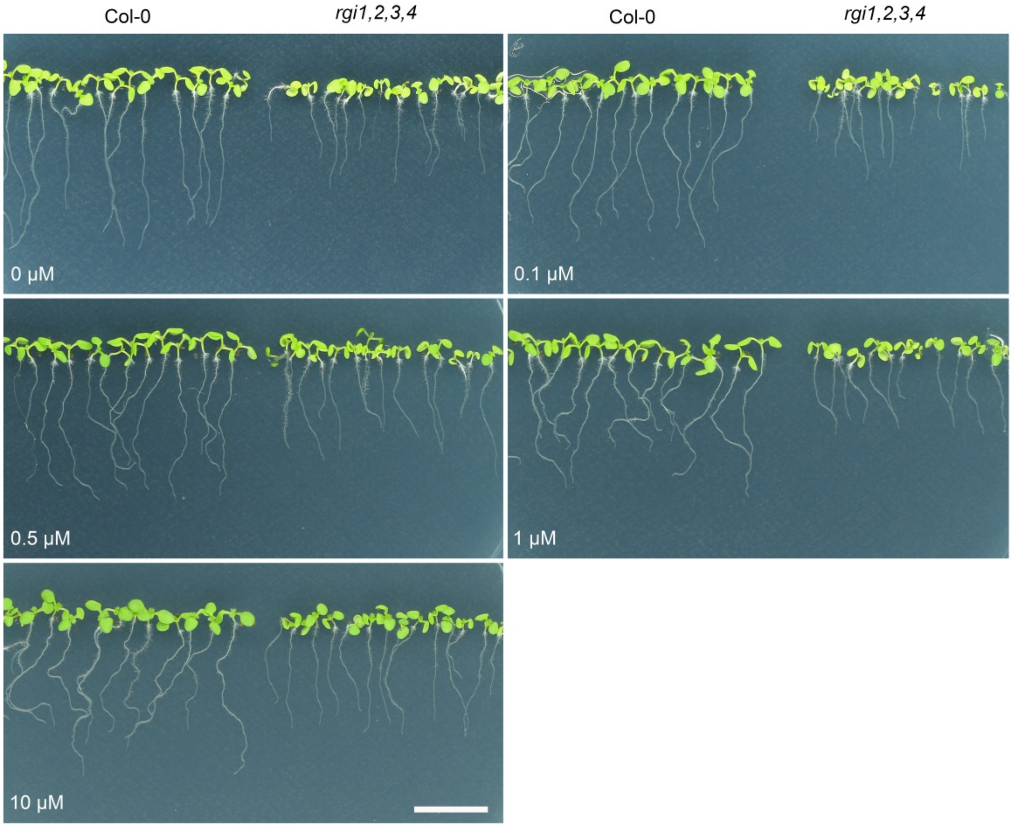

Figure S12. The synthetic peptide derived from MpRGF1 motif triggered a wavy-root phenotype. Six-day-old seedlings grown vertically on MS medium containing the indicated concentration (0, 0.1, 0.5, 1.0, 10.0 μM) of the synthetic MpRGF1 peptide. Scale bar equals to 1 cm.

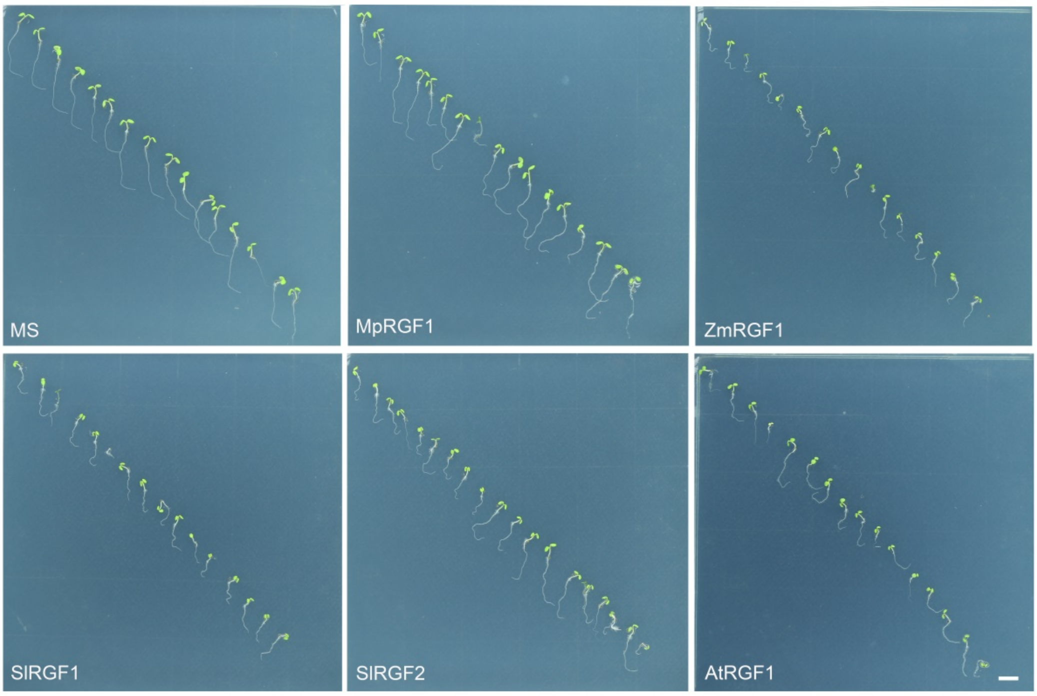

Figure S13. Gravitropic response of wild-type plants following gravistimulation. Three-day-old light-grown seedlings of wild-type plants were germinated on vertical plates and then placed in the dark and reoriented by a 90° angle for 24h. The pictures were taken after the plates were reoriented to its original position after the gravistiulation analysis. Scale bar equals to 5 mm.

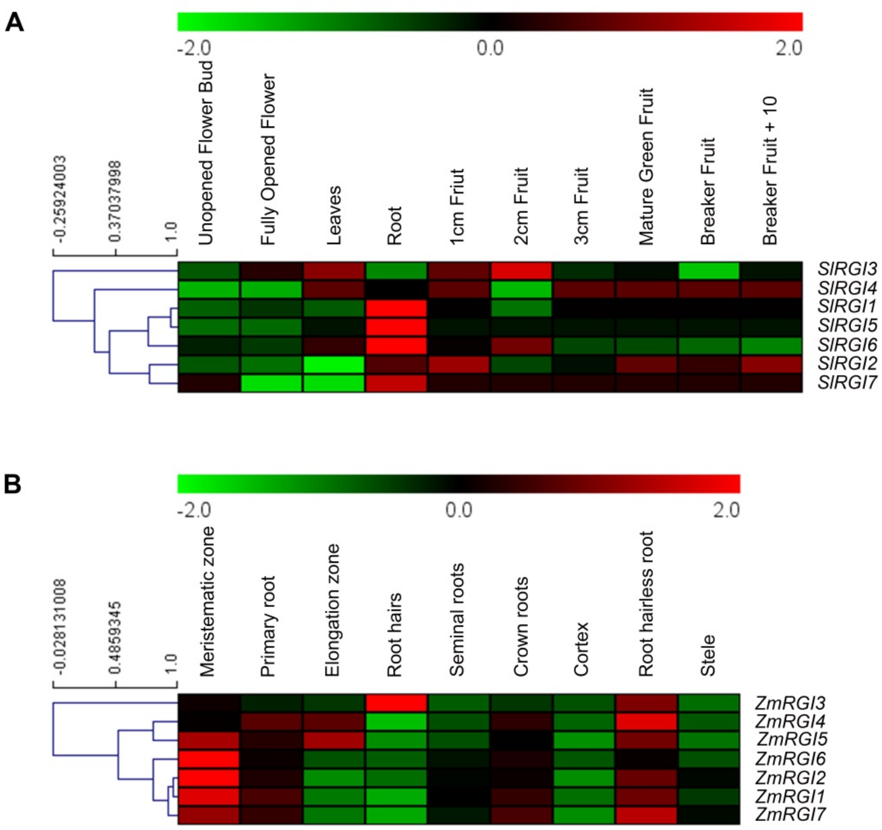

Figure S14. Expression analysis of tomato (**A**) and maize (**B**) *RGI* genes. Heat maps indicate the expression levels of *RGI* genes in different tissues or organs as determined from the e-FP browser. The color scale indicates the expression levels of *RGIs*. Red marks high expression, and green marks low expression.

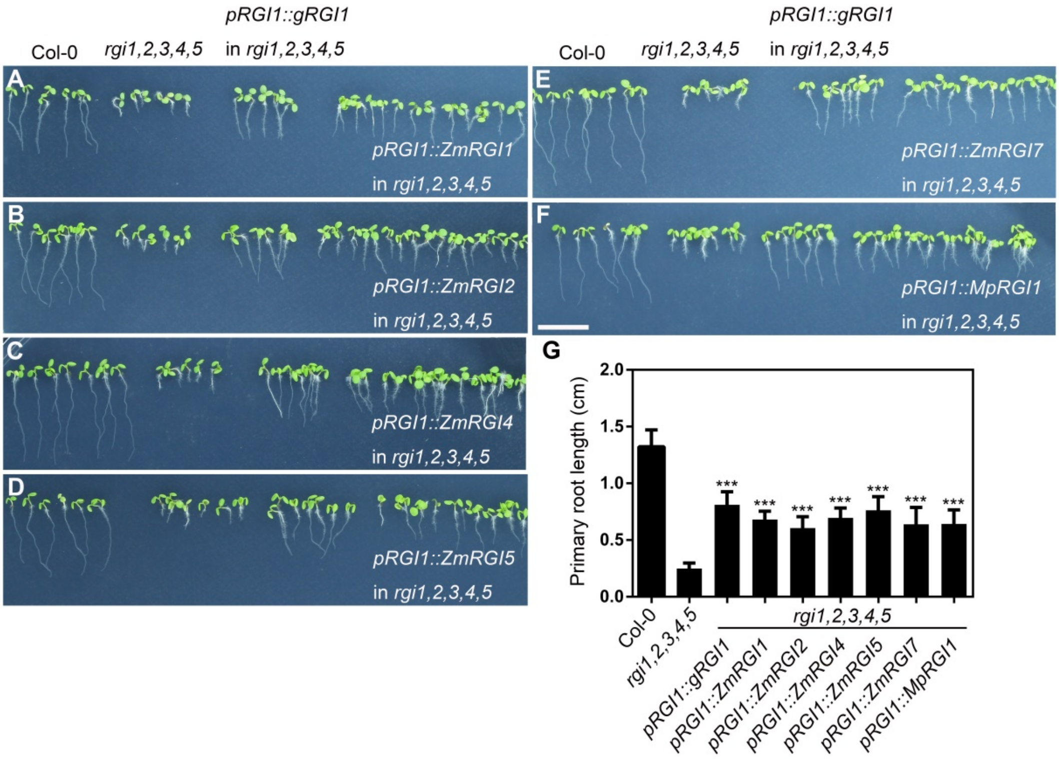

Figure S15. Part of *RGIs* from liverwort and maize can partially rescue the short root phenotype of the Arabidopsis *rgi1,2,3,4,5* quintuple mutant. (**A**-**F**) Seedlings cultured on MS medium for 6 days were used for photographing. (**G**) Statistical results of (**A**-**F**). Student's *t*-tests were performed based on the differences between different transgenic lines and the *rgi1,2,3,4,5* quintuple mutant. "***" means $P < 0.001$. n > 30. Scale bar equals to 1 cm.

Table S4. Segmental duplications in *RGFs*. *Ka* and *Ks* values of each duplicated gene pairs were calculated by using PGDD database (http://chibba.uga.edu/duplication/).

Block NO	Block score	E_value	Locus_1	Locus_2	K_a	K_s	Omega
90	5728	4.00E-19	LOC_Os02g55580	LOC_Os06g08130	0.4053	0.8142	0.497789
80	12731	4.00E-13	LOC_Os02g38330	LOC_Os04g40640	0.3416	0.8844	0.386251
185	974	3.00E-06	Bradi3g16005	Bradi5g23716	0.6225	1.0113	0.615544
51	993	6.00E-13	Bradi1g47720	Bradi3g54310	0.5732	1.1941	0.480027
184	11501	1.00E-10	Bradi3g47791	Bradi5g13666	0.3558	0.9651	0.368666
96	1887	1.00E-21	GRMZM2G050458	GRMZM2G364839	0.2259	0.4544	0.497139
67	2325	6.00E-18	GRMZM2G331770	GRMZM5G846550	0.3319	0.6133	0.541171
61	797	7.00E-49	AC231226.2_FG001	GRMZM2G162271	0.1576	0.2008	0.784861
207	3065	8.00E-11	AC200065.5_FG009	AC202076.3_FG009	0.3832	0.961	0.398751
98	478	4.00E-24	GRMZM2G022255	GRMZM2G041513	0.1656	0.4628	0.357822
413	535	8.00E-14	GRMZM2G074450	GRMZM2G096431	1.2384	1.8081	0.684918
123	237	2.00E-29	Medtr2g450420	Medtr4g116360	0.3544	0.7251	0.48876
194	3214	2.00E-21	Medtr3g095180	Medtr8g092340	0.2949	0.8927	0.330346
149	1449	1.00E-17	Solyc03g114670.2	Solyc06g069350.1	0.4613	1.0803	0.427011
314	795	1.00E-22	Solyc07g021340.2	Solyc12g062690.1	0.0444	0.0426	1.042254
184	8078	4.00E-54	Potri.002G144400	Potri.014G060400	0.1857	0.3881	0.478485
394	317	4.00E-29	Potri.006G060000	Potri.018G119800	0.2485	0.4103	0.605654
386	3395	6.00E-29	Potri.006G060000	Potri.018G115600	0.2481	0.4123	0.601746
323	9577	8.00E-34	Potri.005G087000	Potri.007G077300	0.0682	0.3835	0.177836
21	42231	2.00E-34	Potri.001G123900	Potri.003G109500	0.3507	0.4785	0.732915
292	8042	3.00E-88	Potri.004G112600	Potri.017G102200	0.1643	0.2915	0.563636
31	2286	3.00E-13	AT1G13620	AT2G04025	0.3615	1.0547	0.342751
461	773	4.00E-10	Glyma.04G217600	Glyma.08G145000	0.3667	1.074	0.341434
570	21948	1.00E-22	Glyma.05G186400	Glyma.08G145000	0.0693	0.0352	1.96875
667	879	3.00E-10	Glyma.06G148200	Glyma.08G145000	0.3525	1.0823	0.325695
86	22032	1.00E-91	Glyma.01G210300	Glyma.11G031700	0.0887	0.1031	0.86033
3	5334	1.00E-67	Glyma.01G065000	Glyma.02G122800	0.0605	0.1504	0.402261
1019	377	2.00E-08	Glyma.09G094100	Glyma.13G165000	0.3539	0.894	0.395861
1347	2229	1.00E-82	Glyma.13G165000	Glyma.17G106100	0.045	0.0739	0.608931
979	4817	3.00E-118	Glyma.08G253200	Glyma.18G275800	0.0621	0.07	0.887143
570	21948	5.00E-41	Glyma.05G186300	Glyma.08G144800	0.0173	0.0912	0.189693
630	2547	1.00E-87	Glyma.05G017600	Glyma.17G082400	0.0781	0.1088	0.717831
15	262	8.00E-21	Glyma.01G079000	Glyma.03G031600	0.3414	0.8195	0.416595
18	4069	1.00E-78	Glyma.01G136600	Glyma.03G031600	0.0365	0.0556	0.656475
266	10678	3.00E-47	Glyma.02G079700	Glyma.16G164100	0.0557	0.0889	0.626547
446	28247	4.00E-12	Glyma.04G217600	Glyma.06G148100	0.5326	2.0471	0.260173
554	4057	3.00E-16	Glyma.05G186400	Glyma.06G148100	0.4625	1.2358	0.374251
1346	9256	2.00E-43	Glyma.13G102700	Glyma.17G056400	0.0216	0.0944	0.228814
3	5334	5.00E-31	Glyma.01G067700	Glyma.02G124200	0.1563	0.1503	1.03992

48	640	5.00E-65	Smo_441866	Smo_412527	-1	-1	1
52	1399	1.00E-38	Smo_404292	Smo_438132	0.1954	0.2575	0.758835
0	57645	2.00E-40	16038408	AT1G13620	0.0418	0.1181	0.353937
162	65180	1.00E-55	16035020	AT5G15725	0.068	0.1248	0.544872
93	3443	3.00E-12	16044694	AT5G15725	0.445	1.1455	0.388477
127	6327	3.00E-54	16041430	AT2G03830	0.052	0.1462	0.355677
193	64683	2.00E-33	16038383	AT4G16515	0.0374	0.1204	0.310631
127	6327	2.00E-52	16041583	AT2G04025	0.0584	0.1798	0.324805
79	62910	6.00E-23	16044954	AT3G02240	0.1839	0.3527	0.521406
218	49010	6.00E-40	16037839	AT5G64770	0.0398	0.0945	0.421164
133	60155	6.00E-83	16060007	AT3G30350	0.0616	0.2091	0.294596
218	49010	7.00E-36	16044961	AT5G51451	0.0477	0.0793	0.601513
42	36370	5.00E-25	16049896	AT1G66145	0.2756	0.4408	0.625227
133	60155	3.00E-46	16055107	AT3G60650	0.119	0.2025	0.587654
516	9547	1.00E-15	Bradi5g12825	GRMZM2G364839	0.5307	0.8513	0.6234
547	1229	8.00E-26	Bradi5g23716	GRMZM5G846550	0.3098	0.813	0.381058
364	17767	1.00E-32	Bradi3g47791	GRMZM2G121160	0.2348	0.6345	0.370055
542	6362	2.00E-10	Bradi5g12825	GRMZM2G050458	0.5055	1.0677	0.473448
343	3218	7.00E-08	Bradi3g54310	AC200065.5_FG009	0.4538	1.3581	0.334143
120	5891	3.00E-22	Bradi1g47720	GRMZM2G149126	0.2769	1.1867	0.233336
520	1983	2.00E-12	Bradi5g17935	AC231226.2_FG001	0.6888	1.0037	0.686261
542	6362	9.00E-22	Bradi5g13666	GRMZM2G022255	0.2517	0.6423	0.391873
517	6704	2.00E-12	Bradi5g23716	GRMZM2G331770	0.5174	1.1187	0.462501
516	9547	9.00E-17	Bradi5g13666	GRMZM2G041513	0.2388	0.6057	0.394255
541	7815	5.00E-12	Bradi5g17935	GRMZM2G162271	0.6184	1.0203	0.606096
169	898	4.00E-06	LOC_Os02g44340	AC231226.2_FG001	0.8205	1.3701	0.598861
276	7865	4.00E-17	LOC_Os04g55100	GRMZM2G331770	0.4366	0.8707	0.501436
193	8084	4.00E-09	LOC_Os02g09760	GRMZM2G065781	0.6839	1.6189	0.422447
203	894	7.00E-08	LOC_Os02g09760	GRMZM2G096431	1.0655	1.9751	0.539466
278	2216	4.00E-12	LOC_Os04g46850	AC231226.2_FG001	0.5294	0.6967	0.759868
290	14871	7.00E-12	LOC_Os04g46850	GRMZM2G162271	0.7692	0.9163	0.839463
192	28751	4.00E-15	LOC_Os02g38330	GRMZM2G121160	0.2106	0.5369	0.392252
398	2432	6.00E-36	LOC_Os06g42754	GRMZM2G074450	0.3453	0.6368	0.542242
275	9651	7.00E-18	LOC_Os04g40640	GRMZM2G041513	0.2589	1.0152	0.255024
291	12312	1.00E-21	LOC_Os04g40640	GRMZM2G022255	0.2832	0.9661	0.293137
299	4986	5.00E-15	LOC_Os04g40640	GRMZM2G121160	0.4161	1.0822	0.384495
184	7173	4.00E-13	LOC_Os02g55580	AC200065.5_FG009	0.3532	1.402	0.251926
192	28751	2.00E-14	LOC_Os02g55580	AC202076.3_FG009	0.5734	3.3149	0.172977
397	9356	1.00E-21	LOC_Os06g08130	GRMZM2G149126	0.3676	1.0362	0.354758
275	9651	3.00E-10	LOC_Os04g39230	GRMZM2G364839	0.4953	0.6341	0.781107
291	12312	3.00E-10	LOC_Os04g39230	GRMZM2G050458	0.4903	0.7892	0.621262
420	4050	3.00E-06	Medtr5g012400	Solyc08g078840.2	0.8213	1.9531	0.420511
568	1489	3.00E-08	Medtr8g092340	Solyc04g051420.2	0.6075	1.4562	0.417182

Table S5. K_a/K_s ratio in paralogous pairs of *RGF* genes.

**Paralogous pairs**		**S**	**N**	**omega**	**dN**	**dS**
AT3G02240	AT3G02242	69.4	230.6	1.0198	0.2538	0.2489
AT1G13620	AT2G04025	70.7	241.3	0.4159	0.366	0.88
AT5G51451	AT5G60810	46.3	214.7	0.3074	0.8842	2.8762
Solyc07g021340	Solyc12g062690	26.7	90.3	1.1991	0.046	0.0384
Potri.018G119800	Potri.018G115600	86	250	/	0.0203	0
Potri.002G144400	Potri.014G060400	94.3	310.7	0.4222	0.1725	0.4085
Potri.004G112600	Potri.017G102200	138	510	0.5173	0.1667	0.3223
Potri.005G087000	Potri.007G077300	70.9	157.1	0.28	0.0772	0.2758
Medtr0239s0010	Medtr0377s0010	78.4	269.6	2.3507	0.0303	0.0129
Medtr2g450420	Medtr4g116360	91.1	343.9	0.2663	0.3682	1.3823
Medtr3g095180	Medtr8g092340	46.5	184.5	0.1985	0.2716	1.3683
Bradi1g47720	Bradi4g16256	69.9	179.1	0.5632	0.2237	0.3971
AC200065.5_FG009	AC202076.3_FG009	82.9	217.1	0.397	0.4455	1.1223
GRMZM2G022255	GRMZM2G041513	48.2	164.8	0.3004	0.1614	0.5373
GRMZM2G162271	AC231226.2_FG001	138	285	0.981	0.1518	0.1548
GRMZM2G331764	GRMZM2G331770	87	279	0.8028	0.1561	0.1945
LOC_Os08g27700	LOC_Os10g35444	64.8	181.2	0.5452	0.9728	1.7841
Smo_404292	Smo_438132	111.3	329.7	0.5886	0.1582	0.2688
Smo_412527	Smo_441866	149.3	300.7	/	0.0134	0
PAB00028190	PAB00039641	174.1	593.9	0.9117	0.6068	0.6655

Table S6. CODEML analysis of the selective pattern for each group.

Group	Model	Estimates of Parameters	ln*L*	Positively selected sites (*: P>95%; **: P>99%)
Group A	M0	ω = 0.15604	-1379.201167	
	M1	p: 0.85098 0.14902<br>ω: 0.15573 1.00000	-1376.613018	
	M2	p: 0.85098 0.06369 0.08534<br>ω: 0.15573 1.00000 1.00000	-1376.613018	
	M3	p: 0.06921 0.47954 0.45124<br>ω: 0.00000 0.11073 0.29705	-1360.691265	
	M4	p: 0.07285 0.81949 0.10766<br>0.00000 0.00000<br>ω: 0.00000 0.33333 0.66667<br>1.00000 3.00000	-1370.928583	
	M7(β)	p = 1.39676<br>q = 5.90117	-1362.684933	
	M8<br>(β and ω)	p0 = 0.99999<br>p = 1.39677<br>q = 5.90115<br>(p1 = 0.00001)<br>ω = 1.00000	-1362.68521	
Group B	M0	ω = 0.09588	-978.648429	
	M1	p: 0.59258 0.40742<br>ω: 0.02810 1.00000	-909.764563	
	M2	p: 0.59258 0.20742 0.20000<br>ω: 0.02810 1.00000 1.00000	-909.764563	
	M3	p: 0.29455 0.30078 0.40467<br>ω: 0.00217 0.03732 0.34135	-899.658066	
	M4	p: 0.25704 0.38885 0.00000<br>0.14120 0.21290<br>ω: 0.00000 0.33333 0.66667<br>1.00000 3.00000	-931.876338	
	M7(β)	p = 0.24698<br>q = 0.86113	-900.585808	
	M8<br>(β and ω)	p0 = 0.99999<br>p = 0.24698<br>q = 0.86116<br>(p1 = 0.00001)<br>ω = 1.00000	-900.585811	
Group C	M0	ω = 0.17609	-1109.094047	
	M1	p: 0.46715 0.53285<br>ω: 0.09376 1.00000	-1085.421815	
	M2	p: 0.46715 0.46957 0.06328	-1085.421815	

		ω: 0.09376 1.00000 1.00000	
	M3	p: 0.40782 0.52188 0.07030<br>ω: 0.02622 0.22501 2.31828	-1068.23995
	M4	p: 0.11619 0.64790 0.07120<br>0.16471 0.00000<br>ω: 0.00000 0.33333 0.66667<br>1.00000 3.00000	-1088.508937
	M7(β)	p = 0.70993<br>q = 2.84125	-1070.159295
	M8<br>(β and ω)	p0 = 0.96455<br>p = 0.74711<br>q = 3.57733<br>(p1 = 0.03545)<br>ω = 2.00744	-1070.104709
Group D	M0	ω = 0.06585	-1064.083504
	M1	p: 0.68425 0.31575<br>ω: 0.02606 1.00000	-1002.288397
	M2	p: 0.68425 0.25776 0.05799<br>ω: 0.02606 1.00000 1.00000	-1002.288397
	M3	p: 0.52649 0.26999 0.20352<br>ω: 0.00569 0.07459 0.47408	-972.939018
	M4	p: 0.21050 0.57275 0.00000<br>0.12171 0.09504<br>ω: 0.00000 0.33333 0.66667<br>1.00000 3.00000	-1034.959055
	M7(β)	p = 0.28262<br>q = 1.94574	-972.185966
	M8<br>(β and ω)	p0 = 0.99999<br>p = 0.28262<br>q = 1.94577<br>(p1 = 0.00001)<br>ω = 1.00000	-972.186052
Group E	M0	ω = 0.25965	-1491.032659
	M1	p: 0.24755 0.75245<br>ω: 0.07548 1.00000	-1459.89263
	M2	p: 0.24755 0.61920 0.13325<br>ω: 0.07548 1.00000 1.00000	-1459.89263
	M3	p: 0.17884 0.39618 0.42497<br>ω: 0.01597 0.22809 0.59988	-1442.169088
	M4	p: 0.09081 0.50680 0.05523<br>0.34716 0.00000<br>ω: 0.00000 0.33333 0.66667<br>1.00000 3.00000	-1452.676742

	M7(β)	p = 0.70474<br>q = 1.19610	-1443.81166	
	M8<br>(β and ω)	p0 = 0.99999<br>p = 0.70474<br>q = 1.19612<br>(p1 = 0.00001)<br>ω= 2.78537	-1443.811842	
Group F	M0	ω = 0.14985	-802.69092	
	M1	p: 0.62326 0.37674<br>ω: 0.06444 1.00000	-767.36782	
	M2	p: 0.62326 0.24761 0.12913<br>ω: 0.06444 1.00000 1.00000	-767.36782	
	M3	p: 0.22069 0.41531 0.36400<br>ω: 0.00330 0.09225 0.66676	-756.729738	
	M4	p: 0.15792 0.52911 0.00000<br>0.20678 0.10619<br>ω: 0.00000 0.33333 0.66667<br>1.00000 3.00000	-768.393313	
	M7(β)	p = 0.36409<br>q = 0.91021	-759.044137	
	M8<br>(β and ω)	p0 = 0.89997<br>p = 0.44465<br>q = 1.75878<br>(p1 = 0.10003)<br>ω= 1.76501	-758.608163	1 S 10 G
Group G	M0	ω = 0.00224	-720.707068	
	M1	p: 0.69156 0.30844<br>ω: 0.04934 1.00000	-714.234832	
	M2	p: 0.66376 0.12221 0.21403<br>ω: 0.08010 1.00000 63.89464	-713.777233	1 M 4 A 24 I 26 G<br>48 Q 49 E
	M3	p: 0.24054 0.52264 0.23682<br>ω: 0.00000 0.15078 38.31321	-710.435893	
	M4	p: 0.26266 0.53303 0.00000<br>0.00000 0.20431<br>ω: 0.00000 0.33333 0.66667<br>1.00000 3.00000	-712.51945	
	M7(β)	p = 0.44165<br>q = 1.47164	-713.124319	
	M8	p0 = 0.77716<br>p = 0.63805<br>q = 3.81979<br>(p1 = 0.22284)<br>ω = 48.58975	-711.309555	1 M 4 A 24 I<br>26 G 48 Q 49 E

Group H	M0	ω = 0.07936	-983.364551	
	M1	p: 0.75037 0.24963<br>ω: 0.07634 1.00000	-954.449406	
	M2	p: 0.12499 0.66360 0.21141<br>ω: 0.00000 1.00000 4.09212	-990.918416	2 T * 6 L * 14 W **<br>15 K ** 17 R
	M3	p: 0.12438 0.62191 0.25371<br>ω: 0.00000 0.05357 0.39521	-938.803268	
	M4	p: 0.12493 0.64243 0.00000<br>0.14623 0.08641<br>ω: 0.00000 0.33333 0.66667<br>1.00000 3.00000	-957.914964	
	M7(β)	p = 0.57801<br>q = 4.15942	-939.496468	
	M8	p0 = 0.99999<br>p = 0.57801<br>q = 4.15939<br>(p1 = 0.00001)<br>ω = 1.00000	-939.496569	
Group I	M0	ω = 0.26360	-1443.583719	
	M1	p: 0.59699 0.40301<br>ω: 0.15978 1.00000	-1420.199687	
	M2	p: 0.59699 0.29115 0.11187<br>ω: 0.15978 1.00000 1.00000	-1420.199687	
	M3	p: 0.10035 0.46847 0.43117<br>ω: 0.00000 0.15175 0.59106	-1410.652294	
	M4	p: 0.10967 0.63778 0.00000<br>0.25255 0.00000<br>ω: 0.00000 0.33333 0.66667<br>1.00000 3.00000	-1417.469654	
	M7(β)	p = 0.78763<br>q = 1.59246	-1412.066059	
	M8	p0 = 0.99999<br>p = 0.78764<br>q = 1.59250<br>(p1 = 0.00001)<br>ω = 1.00000	-1412.066078	

Table S7. Likelihood ratio test of positive selection under site models for RGFs in plants.

Group	2L/M3 vs. M0	2L/M2 vs. M1	2L/M8 vs. M7
A	37.0198 *	0	0.00056
B	157.980726 *	0	0.000006
C	81.7082 *	0	0.10918
D	182.288964 *	0	0.000172
E	97.72714 *	0	0.00036
F	91.922364 *	0	0.871948
G	20.54235 *	0.915198	3.629528
H	89.122566 *	72.93802 *	0.000202
I	65.86286 *	0	0.00004

* indicates signicant at $p < 0.001$ level.

Publisher's Note: MDPI stays neutral with regard to jurisdictional claims in published maps and institutional affiliations.

© 2020 by the authors. Submitted for possible open access publication under the terms and conditions of the Creative Commons Attribution (CC BY) license (http://creativecommons.org/licenses/by/4.0/).
